# Transferrin receptor 1 is required for efficient hepatitis E virus production

**DOI:** 10.1371/journal.ppat.1014484

**Published:** 2026-08-18

**Authors:** Nathalie Da Silva, Angela Pollán, Luca Truscello, Manfredo Quadroni, Darius Moradpour, Jérôme Gouttenoire

**Affiliations:** 1 Division of Gastroenterology and Hepatology, Lausanne University Hospital and University of Lausanne, Lausanne, Switzerland; 2 Protein Analysis Facility, University of Lausanne, Lausanne, Switzerland; Princeton University, UNITED STATES OF AMERICA

## Abstract

Hepatitis E virus (HEV) infection is a major cause of acute viral hepatitis worldwide. HEV is a positive-strand RNA virus encoding three open reading frames (ORFs). The ORF3 protein is a small membrane-associated protein essential for viral particle secretion; however, its precise role in the viral life cycle remains incompletely understood. Here, we performed immunoprecipitation followed by mass spectrometry to identify host proteins interacting with the HEV ORF3 protein. Candidate interactors were validated by co-immunoprecipitation, confirming physical interactions between ORF3 and cysteine-rich and transmembrane domain-containing protein 1 (CYSTM1), Ras-related protein Rab24, and transferrin receptor 1 (TfR1). Confocal microscopy demonstrated colocalization of all three host factors with ORF3 protein. Gene silencing and knockout revealed that each protein contributes to virus production, with TfR1 depletion producing the most pronounced effect. In cells harboring replicating HEV, TfR1 colocalized with the ORF2 (capsid) and ORF3 proteins at Rab11A-positive recycling endosomes. Silencing of TfR1 in primary human hepatocytes, followed by HEV RNA transfection or infection, confirmed its role in virus production, particularly in the assembly of infectious particles, consistent with its colocalization with the HEV ORF2 protein. Collectively, our proteomics-based analysis identifies TfR1, along with CYSTM1 and Rab24, as novel ORF3-interacting host factors required for efficient production of infectious HEV. These findings provide new insights into the role of ORF3 protein in viral assembly and highlight TfR1 as a key host factor in the HEV life cycle.

## Author summary

Hepatitis E virus (HEV) infection is a leading cause of acute hepatitis and jaundice worldwide. HEV is a positive-strand RNA virus that, despite being classically considered non-enveloped, exploits the exosomal pathway for its release. The HEV ORF3 protein plays a critical role in this process, but how it functions remains incompletely understood. Using a proteomics-based approach, we identified three previously unknown host interaction partners of the ORF3 protein, i.e., the cysteine-rich protein CYSTM1, the Ras-related protein Rab24, and the transferrin receptor TfR1. We show that TfR1 localizes together with the viral ORF2 and ORF3 proteins at recycling endosomes and is required for efficient HEV production, most likely during virion assembly, including in primary human hepatocytes. Our findings provide new insights into how HEV exploits host cell pathways for assembly and release and identify TfR1 as an important host factor in the HEV life cycle.

## Introduction

Hepatitis E virus (HEV) infection is a leading cause of acute hepatitis worldwide, with waterborne human-to-human transmission of genotypes (gt) 1 and 2 occurring in resource-limited regions. In contrast, infections with HEV gt 3 and 4 have emerged as a primarily porcine zoonoses in industralized countries, with mainly foodborne transmission and seroprevalence rates ranging from 20 to 86% in some regions [1-3]. Although HEV gt 3 infection is usually self-limited and often asymptomatic or associated with only mildly symptoms, it can cause severe hepatitis, acute-on-chronic liver failure, as well as neurological, renal and other extrahepatic manifestations in some individuals. Moreover, HEV infection may persist in immunocompromised individuals and cause rapidly progressive liver disease [1,2]. Currently, treatment of chronic hepatitis E relies on reduction of immunosuppression when feasible and administration of ribavirin, a broad-spectrum antiviral.

HEV, a member of the *Hepeviridae* family [4], has a 7.2-kb positive-strand RNA genome which encodes three proteins: the multifunctional ORF1 protein containing the catalytic domains required for viral genome replication, the ORF2 capsid protein, and the small ORF3 protein required for viral particle secretion [5,6]. Despite recent progress, many aspects of the HEV life cycle remain poorly understood, including the molecular functions of viral proteins and their interactions with host factors.

A distinctive feature of HEV is the secretion of viral particles within host-derived membranes, similar to hepatitis A virus [7]. As a result, intracellular virions are non-enveloped, whereas extracellular particles are ‘quasi-enveloped.’ Circulating virions in the bloodstream are wrapped in membranes, whereas after secretion into bile, the viral particles likely lose this membrane through action of bile salts and appear non-enveloped in feces (reviewed in [8]). Viral egress involves the multivesicular body pathway and is dependent on the ORF3 protein ([9] and reviewed in [10]). This process relies on a conserved PSAP late domain motif within ORF3 that enables recruitement of the endosomal sorting complex required for transport (ESCRT) [11-14]. In particular, ORF3 protein interacts with the ESCRT-I component tumor susceptibility gene 101 protein (Tsg101) to mediate secretion of infectious particles [11,12]. ORF3 protein is also essential for apical release of HEV in polarized cells and for virus secretion into bile in infected animals [15].

Several biochemical features of ORF3 protein have been linked to its role in viral secretion. A phosphorylated form of ORF3 has been reported to facilitate interaction with the capsid protein [16]. In addition, ORF3 protein has been reported to exhibit ion channel activity that is important for virus secretion, although the underlying mechanisms remain unclear [17]. Our group previously demonstrated that the HEV ORF3 protein forms membrane-associated oligomers and undergoes posttranslational S-acylation on eight highly conserved cysteine residues located in its N-terminal region [18]. These modifications, which regulate protein stability, membrane association, and subcellular localization, are essential for ORF3-mediated viral secretion. Beyond its role in viral egress, ORF3 protein has also been implicated in modulation of host antiviral responses [15,19] and in the regulation of cellular lipid metabolism, promoting lipid droplet biogenesis [20].

In the present study, we used specific recombinant antibodies to immunoprecipitate ORF3 protein from HEV replicating cells in order to identify novel host interaction partners. Mass spectrometry (MS) followed by validation through co-immunoprecipitation and confocal microscopy led to the identification of three previously unrecognized host factors interacting with ORF3 protein. Functional assays employing siRNA silencing and CRISPR-Cas9 knockout demonstrated their importance in virus production. In particular, we show that transferrin receptor 1 (TfR1) colocalizes with HEV ORF3 and ORF2 proteins at recycling endosomes and contributes to virus assembly. These findings were further confirmed in primary human hepatocytes (PHH).

## Results

### Identification of host proteins interacting with the HEV ORF3 protein

**Fig 1:**
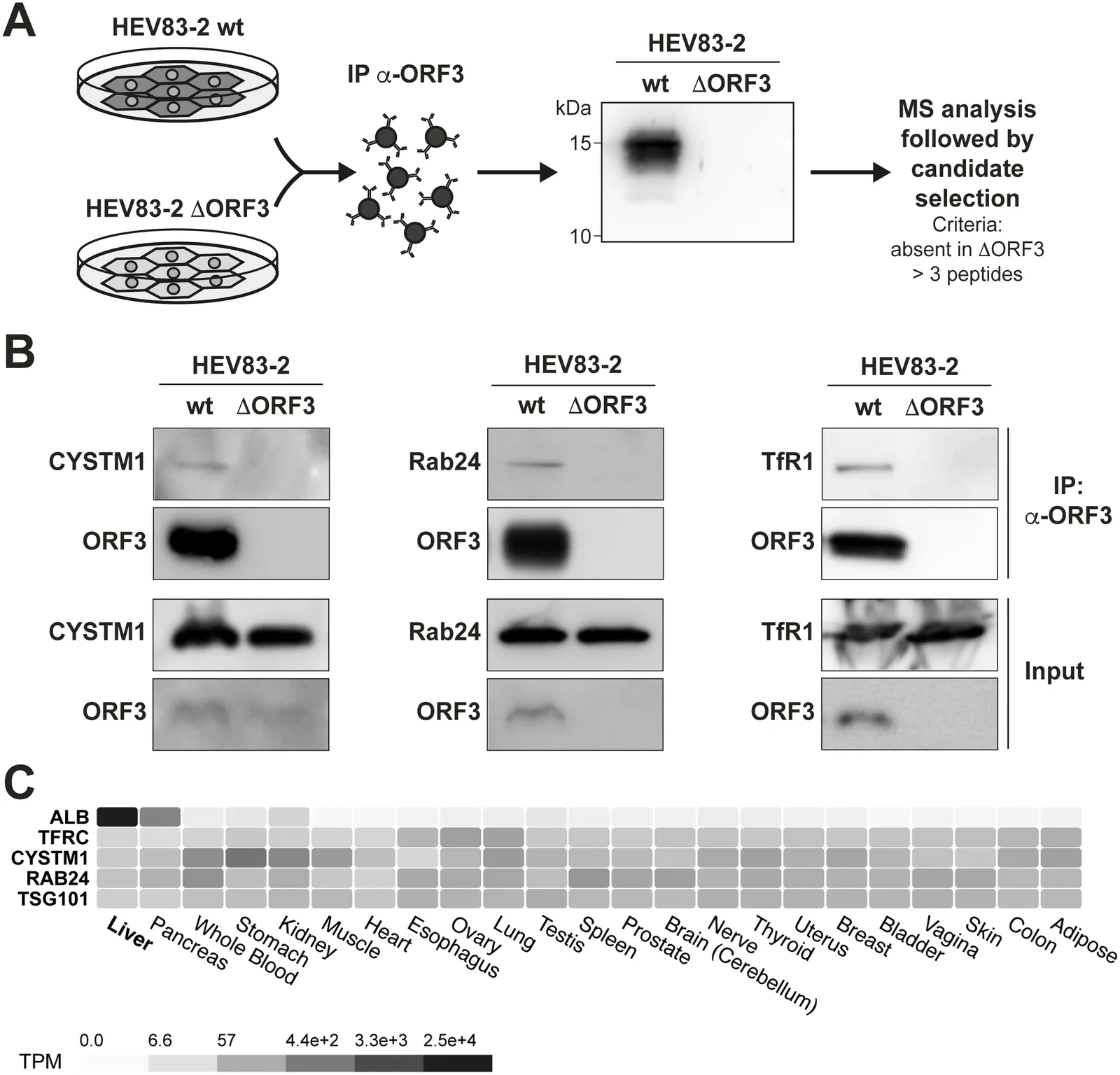
Proteomic identification of novel host interaction partners of the HEV ORF3 protein. (A) Experimental workflow. *In vitro*-transcribed RNA corresponding to the full-length HEV83-2 genome, either wild-type (wt) or ΔORF3, was electroporated into Hep293TT human hepatoblastoma cells. Five days post-electroporation, cells were harvested and protein lysates subjected to immunoprecipitation using a mixture of anti-ORF3 recombinant antibodies #198 and #200 [18] coupled to magnetic beads. A small fraction of the immunoprecipitate was analyzed by SDS-PAGE and immunoblotting, while the remaining fraction was subjected to mass spectrometry (MS). Candidate ORF3-interacting proteins were selected by comparing MS data obtained from wt and ∆ORF3 samples, applying the following criteria: absence in the ΔORF3 condition and identification by at least three unique peptides. (B) Validation of the interactions between HEV ORF3 protein and CYSTM1, Rab24 and TfR1. Hep293TT_AD cells stably expressing FLAG-tagged CYSTM1, Rab24 or TfR1 were electroporated with wt or ΔORF3 full-length HEV83-2 RNA. Protein lysates were subjected to ORF3 immunoprecipitation as described in (A). Lysates (Input) and immunoprecipitates (IP) were analyzed by SDS-PAGE and immunoblotting using mouse anti-FLAG M2 or rabbit anti-ORF3 antibodies. (C) Tissue expression profiles of the identified factors. Data were retrieved from the Adult Genotype Tissue Expression (GTEx) Project (https://gtexportal.org), a comprehensive, publicly available resource of human transcriptomic data. Expression levels across tissues are shown as shades of grey corresponding to transcripts per million (TPM).

To identify host proteins interacting with the HEV ORF3 protein, we performed a proteomic analysis of samples immunoprecipitated with anti-ORF3 antibodies from lysates of Hep293TT human hepatoblastoma cells harboring replicating HEV [18]. Full-length RNA transcribed from wild-type (wt) or ΔORF3 HEV83–2 (gt 3) constructs was electroporated into Hep293TT cells. Because ΔORF3 HEV RNA replicates similarly to the wt and expresses all viral factors except ORF3, it provides a robust negative control for immunoprecipitation experiments. Five days post-electroporation, cell lysates were subjected to immunoprecipitation using a mixture of recombinant anti-ORF3 antibodies #198 and #200 [18], followed by MS. Candidate interactors were selected by comparing MS results obtained from wt and ΔORF3 samples using the following criteria: (i) absence in the ΔORF3 sample and (ii) detection by at least three peptides (Fig 1A). Twenty-two candidates met these criteria, including Tsg101, a known interactor of ORF3, among the top hits (S2 Table). Gene ontology analysis revealed significant enrichment in proteins involved in the regulation of extracellular exosome assembly, consistent with the established role of ORF3 protein in HEV secretion *via* the exosomal pathway.

To validate the MS results, candidates were individually tested for interaction with ORF3 protein by co-immunoprecipitation from Hep293TT_AD cells harboring replicating HEV, followed by immunoblot analysis. Hep293TT_AD cells were adapted from Hep293TT cells and remain permissive to HEV infection while exhibiting improved growth as well as tolerance to siRNA transfection and lentivirus transduction. Stable Hep293TT_AD cells expressing FLAG-tagged candidate proteins were generated by lentiviral transduction followed by blasticidin selection and subsequently transfected with wt or ΔORF3 HEV83–2 genomes. Using immunoprecipitation conditions similar to those employed in the initial pull-down, we confirmed the previously reported interaction between ORF3 protein and Tsg101 (S1 Fig) and validated interactions with cysteine-rich and transmembrane domain-containing protein 1 (CYSTM1), Ras-related protein Rab24, and transferrin receptor 1 (TfR1) (Fig 1B). RNA sequencing data from the Adult Genotype Tissue Expression (GTEx) Project indicate that these proteins, including Tsg101, are ubiquitously expressed rather than hepatocyte-specific (Fig 1C).

**Fig 2:**
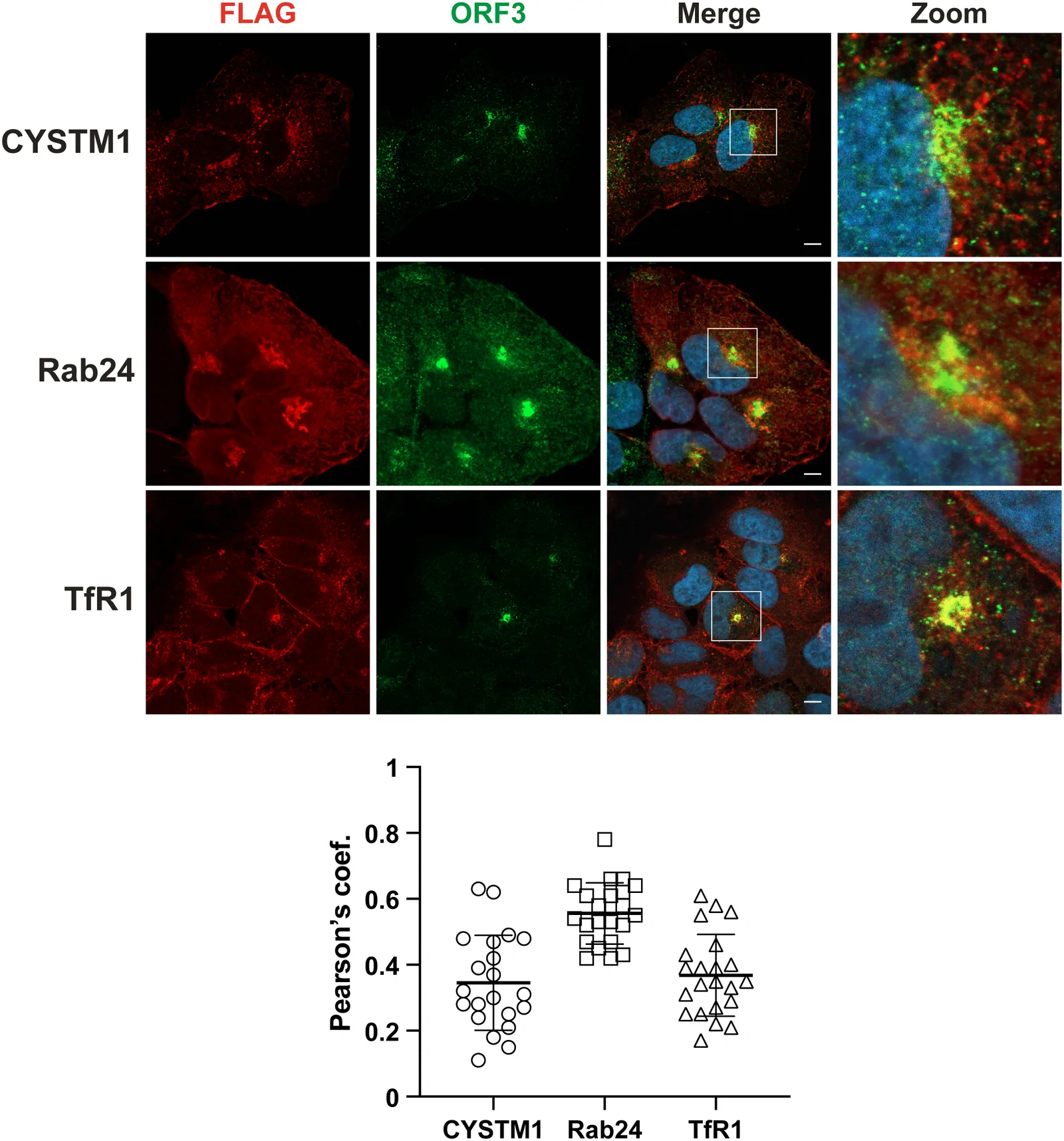
HEV ORF3 protein and newly identified host partners colocalize in the cytoplasm of hepatoblastoma cells. Hep293TT_AD cells stably expressing FLAG-tagged CYSTM1, Rab24 or TfR1 were electroporated with wild-type (wt) or ΔORF3 full-length HEV83-2 RNA and cultured on glass coverslips for 5 days. Following fixation, cells were subjected to immunofluorescence with mouse anti-FLAG M2 (red) and rabbit anti-ORF3 (green). Nuclei were counterstained with DAPI (blue). Representative images, including merged and enlarged views, are shown. Scale bars, 10 µm. Pearson’s coefficient was determined in at least 20 cells in each condition and results are shown as a graph in the lower panel.

To corroborate these findings, immunofluorescence analyses were performed in cells expressing FLAG-tagged candidate proteins. As shown in Fig 2, confocal microscopy revealed a high degree of colocalization between HEV ORF3 protein and the three newly identified host factors, with nearly complete colocalization in perinuclear foci observed for TfR1. Together, these results indicate that ORF3 protein interacts with CYSTM1, Rab24 and TfR1 in the context of HEV genome replication.

### Gene silencing reveals a role for TfR1 in HEV production

**Fig 3:**
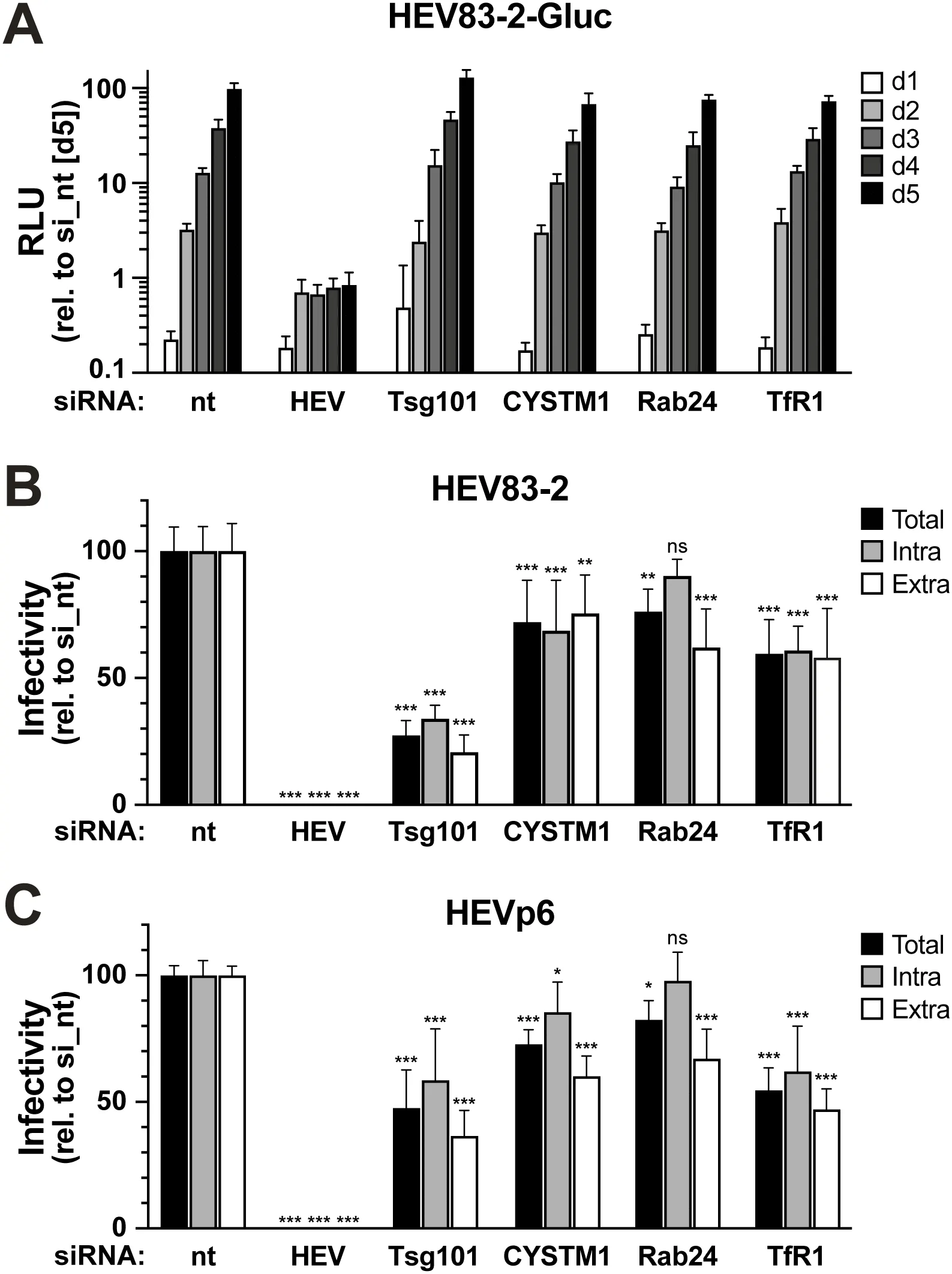
siRNA-mediated knockdown of CYSTM1, Rab24 and TfR1 reduces HEV production. (A) Effect of siRNA-mediated knockdown of newly identified ORF3 interaction partners on HEV replication assessed by a Gaussia luciferase (Gluc) replicon. Hep293TT_AD cells were electroporated with *in vitro*-transcribed HEV83–2 Gluc replicon RNA and transfected with siRNAs targeting the HEV genome, Tsg101, CYSTM1, Rab24 or TfR1, or with non-targeting siRNA (nt) as control. Culture supernatants were collected daily over 5 days, with daily medium replacement. Relative light units (RLU) in culture supernatants were measured at days 1–5 (d1-5) post-electroporation. Data represent the mean of three independent experiments. (B, C) Effect of siRNA-mediated knockdown of the identified host factors on HEV particle production. Hep293TT_AD cells were electroporated with *in vitro*-transcribed full-length HEV83–2 RNA (B, n=6) or p6 RNA (C, n=7) and transfected with siRNAs targeting the HEV genome, Tsg101, CYSTM1, Rab24 or TfR1, or with nt siRNA as control. Intra- and extracellular infectivities were determined by focus-forming unit (FFU) assays 5 days post-electroporation. Total, intracellular and extracellular infectivities are shown relative to the nt control. Statistical analysis was performed using two-way ANOVA followed by Dunnett’s multiple comparison test. ns, not significant; * P< 0.05; ** P< 0.005; *** P< 0.0005.

To investigate whether the identified ORF3-interacting proteins contribute to HEV RNA replication, Hep293TT_AD cells were electroporated with subgenomic HEV Gaussia luciferase (Gluc) reporter replicons derived from the HEV83–2 or the p6 clone, followed by transfection with siRNA pools targeting each host factor. Culture supernatants were collected over 5 days and luciferase activity was quantified as a measure of viral replication. As shown in Figs 3A and S2, silencing of CYSTM1, Rab24 and TfR1 did not affect HEV RNA replication. Of note, Tsg101 silencing consistently resulted in a 2- to 4-fold increase in Gluc activity from both HEV83–2 (Fig 3A) and p6 replicons (S2 Fig).

Given the absence of an effect of CYSTM1, Rab24 and TfR1 silencing on HEV RNA replication, we next examined the impact of gene silencing on virus production. Intracellular and extracellular compartments were collected 5 days after electroporation of full-length HEV83–2 or p6 RNA. Tsg101 silencing markedly reduced infectious HEV levels in both compartments (Figs 3B and 3C), confirming its role in particle secretion and showing a potential implication in virion assembly. Knockdown of CYSTM1 and Rab24 reduced total virus production by approximately 27% and 20%, respectively (Figs 3B and 3C). Rab24 silencing had a more pronounced effect on extracellular infectivity, suggesting an impact on virus secretion (Fig 3B and 3C). The strongest effect was observed following TfR1 silencing, with total infectious titers reduced by 40% and 45% for HEV83–2 and p6, respectively (Figs 3B and 3C). Similar reductions in intra- and extracellular infectivity suggest that TfR1 primarily affects virion assembly.

**Fig 4:**
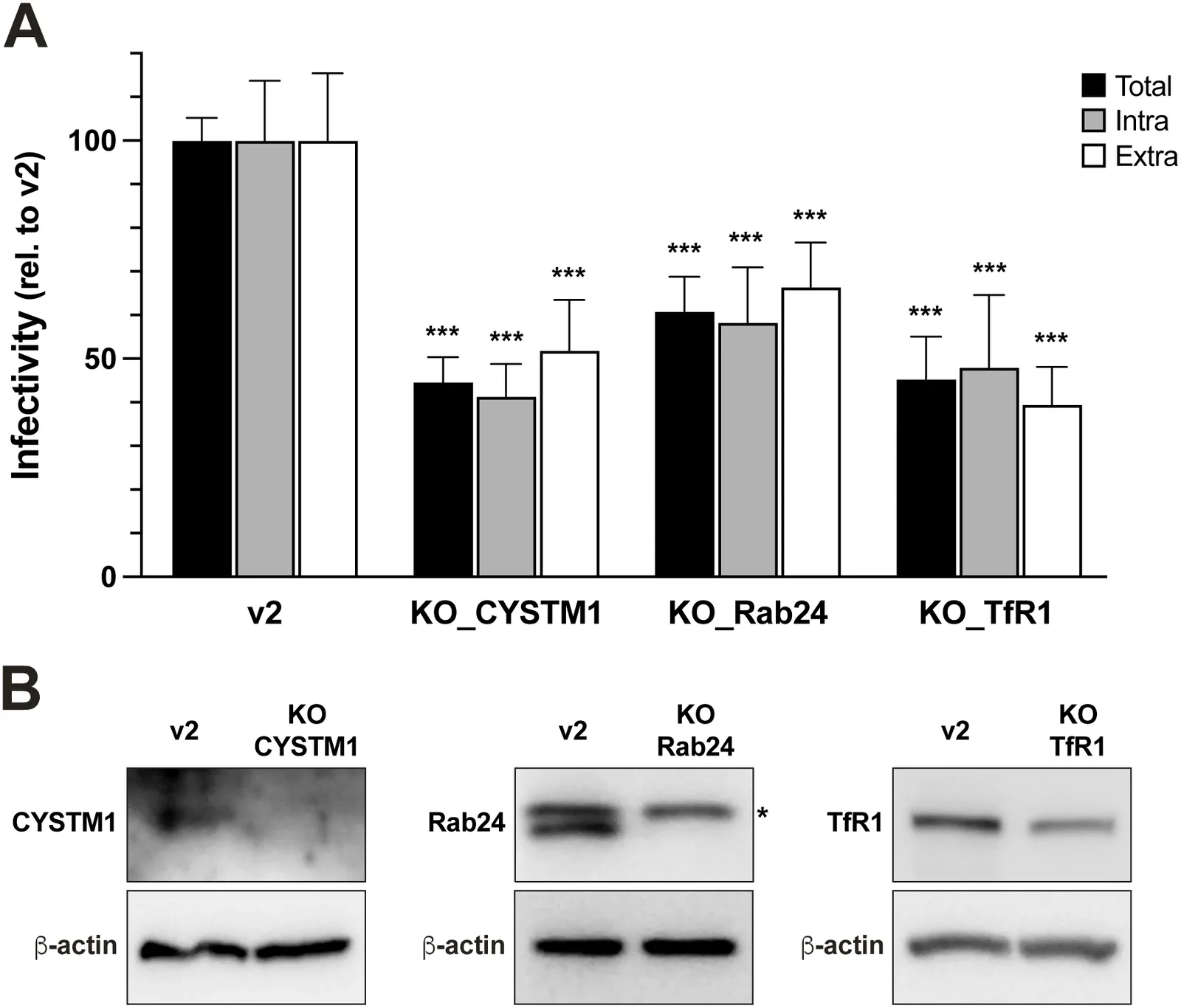
HEV particle production is reduced in CYSTM1, Rab24 and TfR1 knockout cell populations. (A) Effect of gene knockout (KO) on HEV particle production. KO_CYSTM1, KO_Rab24, KO_TfR1 and control cells transduced with the empty LentiCRISPRv2 vector (v2) were electroporated with HEV p6 RNA. Five days later, intra- and extracellular infectivity was measured by focus-forming unit (FFU) assay and expressed relative to the v2 control. Data represent the mean of six values from an experiment performed in triplicate. Statistical analysis was performed using two-way ANOVA followed by Dunnett’s multiple comparison test. *** P < 0.0001. (B) Analysis of protein expression in KO cell populations. Under the same experimental conditions as in (A), cell lysates were collected and protein expression was analyzed by SDS-PAGE and immunoblot using mouse monoclonal antibodies against CYSTM1, Rab24, TfR1 and β-actin. The asterisk denotes a non-specific band.

To confirm these observations using an independent loss-of-function approach, CRISPR-Cas9 knockout (KO) cell populations were generated individually for each of the three genes using the LentiCRISPRv2 vector, which enables lentiviral delivery of Cas9 nuclease and a single guide RNA. Following one week of selection, transduced Hep293TT_AD cells were electroporated with full-length HEV p6 RNA. Five days later, intra- and extracellular compartments were collected for analysis. Infectivity was significantly reduced in all KO cell populations compared to control cells transduced with the empty vector (v2), with reductions ranging from 35 to 60% (Fig 4A). Notably, no significant differences were observed between intra- and extracellular infectivities. The absence of detectable CYSTM1 and Rab24 expression confirmed efficient KO in the corresponding cell populations (Fig 4B). Although TfR1 expression was makedly reduced, complete KO was not achieved in the KO_TfR1 cell population (Fig 4B). Nevertheless, infectivity was reduced more than 50%, further underscoring the importance of TfR1 in virus production. Taken together, these loss-of-function experiments consistently demonstrate importance of all three host factors in infectious virion production.

Given the strong impact of TfR1 expression silencing or KO on virus production, we further investigated this host factor. The interaction between endogenous TfR1 and ORF3 protein was confirmed by co-immunoprecipitation from Hep293TT_AD cells electroporated with wt but not ΔORF3 HEV83–2 genomes (S3A Fig). Colocalization of endogenous TfR1 with ORF3 protein was further confirmed by immunofluorescence in Hep293TT_AD and Huh-7-derived S10-3 cells harboring replicating HEV83–2 or p6 genomes (S3B, S4A and S5A Figs). Consistent with these findings, TfR1 silencing also reduced HEV production in S10-3 cells (S6 Fig).

### TfR1 colocalizes with HEV ORF3 and ORF2 proteins at recycling endosomes

**Fig 5:**
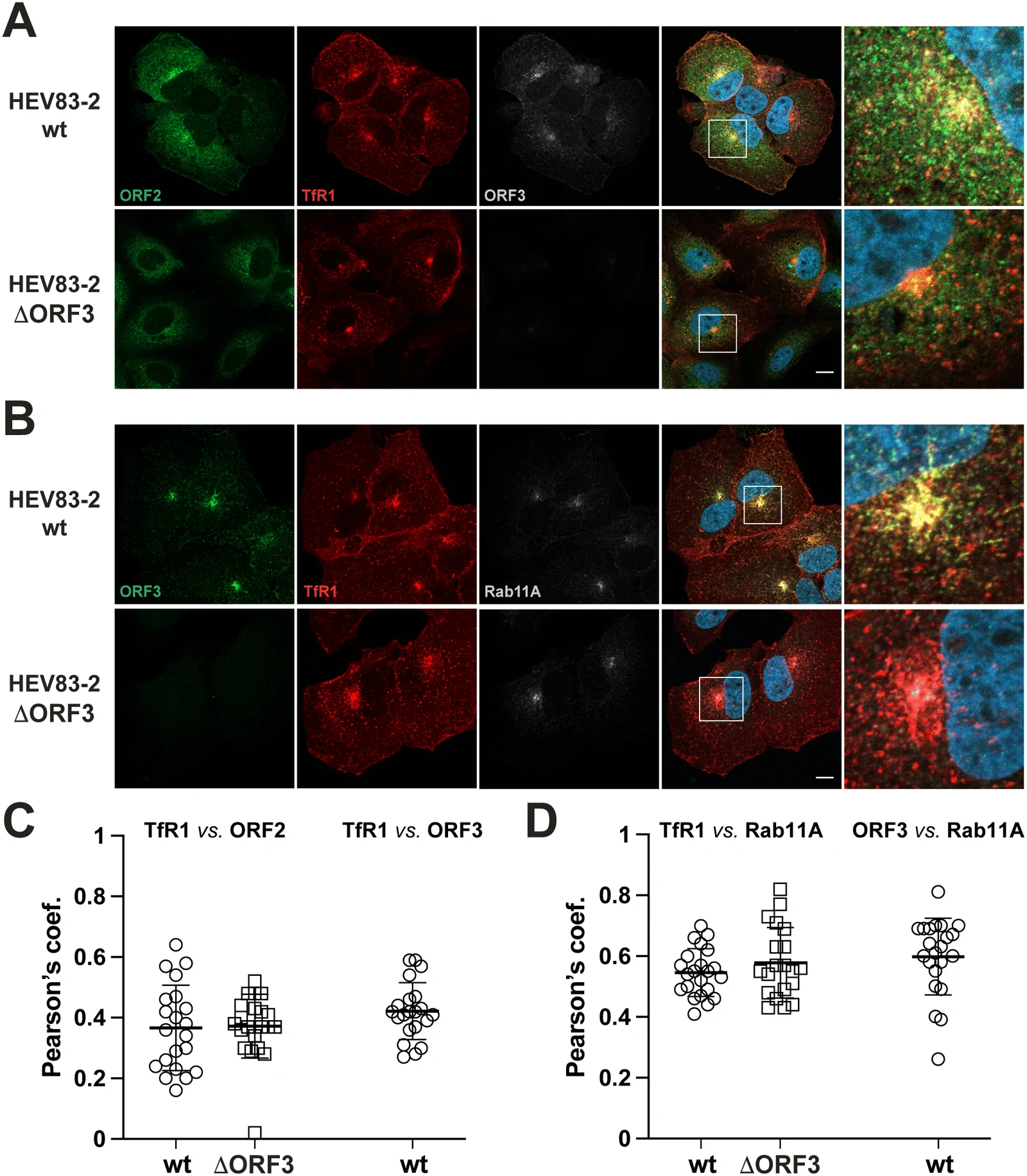
TfR1 colocalizes with HEV ORF2 and ORF3 proteins at recycling endosomes. (A, B) Immunofluorescence detection of HEV ORF2 and ORF3 proteins. Hep293TT_AD cells stably expressing mCherry-tagged TfR1 were electroporated with *in vitro*-transcribed HEV83-2 RNA, either wild-type (wt) or ΔORF3. Five days post-electroporation, cells grown on glass coverslips were fixed and stained with (A) rabbit polyclonal anti-ORF2 antibody (green) and mouse monoclonal anti-ORF3 antibody (grey), or (B) mouse monoclonal anti-ORF3 antibody (green) and rabbit monoclonal anti-Rab11A antibody (grey). TfR1 was visualized by intrinsic mCherry fluorescence (red). Nuclei were counterstained with DAPI (blue). Representative images, including merged and enlarged views (white squares), are shown. Scale bars, 10 µm. (C, D) Colocalization between (C) TfR1 and ORF2 or ORF3 and (D) Rab11A and TfR1 or ORF3 was evaluated, in at least 20 cells, by determination of Pearson’s coefficient.

To further investigate the subcellular localization of TfR1, mCherry-tagged TfR1 was expressed by lentiviral transduction. Tagged TfR1 localized to the plasma membrane and cytoplasm, including perinuclear foci similar to endogenous TfR1 (Figs 2 and S3-S5). Both HEV ORF2 and ORF3 proteins colocalized with TfR1 at these structures across different cell systems and viral clones (Figs 5A and S3-S5). Of note, the subcellular distribution of TfR1 and ORF2 protein was unchanged in cells replicating the HEV83–2 ΔORF3 genome, indicating that ORF3 protein is not required for their localization (Figs 5A and 5C). Given that TfR1 traffics through recycling endosomes, Rab11A staining was performed, revealing strong colocalization of ORF3 protein with TfR1 at Rab11A-positive recycling endosomes appearing as perinuclear foci (Figs 5B and 5D).

### Confirmation of the role of TfR1 in HEV production in primary human hepatocytes

**Fig 6:**
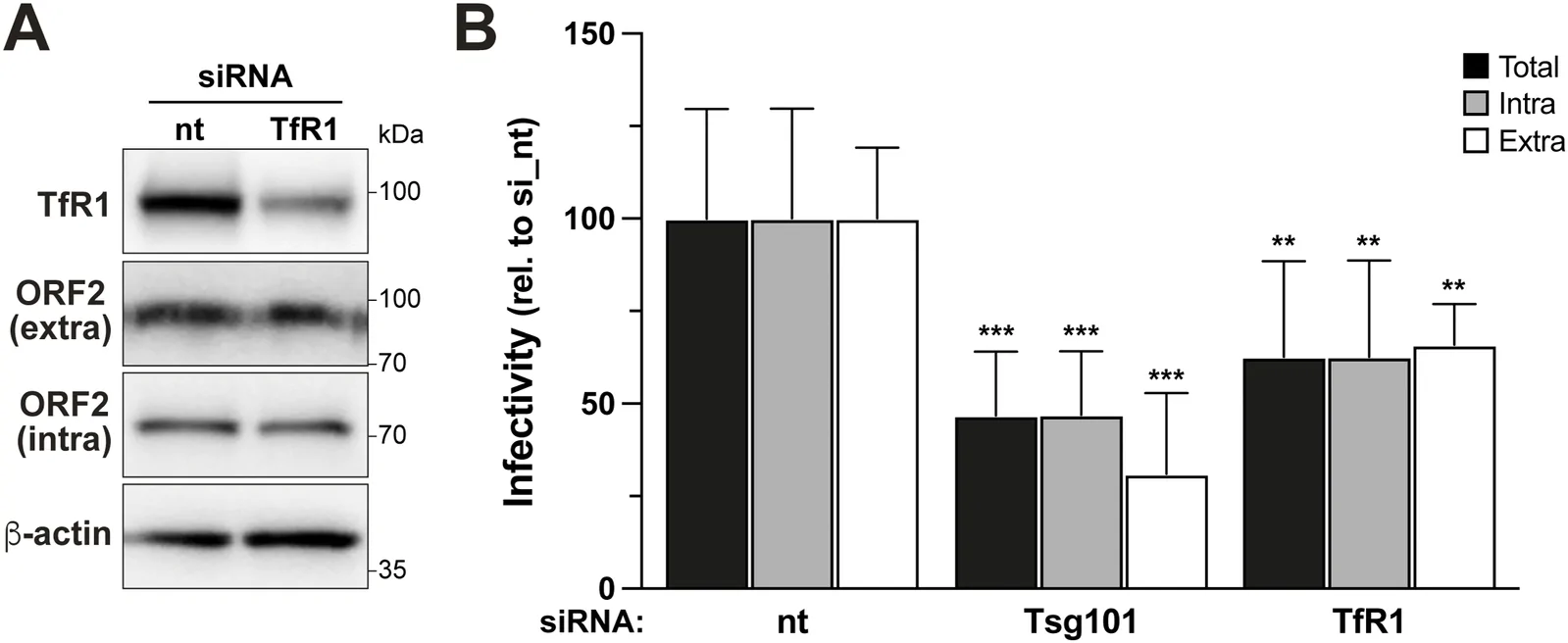
TfR1 knockdown in primary human hepatocytes reduces HEV production. (A) Analysis of protein expression after siRNA-mediated TfR1 knockdown in primary human hepatocytes (PHH). Cells were transfected with siRNA targeting TfR1 or with non-targeting (nt) siRNA as control and, on the following day, transfected with *in vitro*-transcribed full-length HEV p6 RNA and treated with the Jak inhibitor baricitinib (2 µM). Three days after HEV RNA transfection, cell lysates and supernatants were collected. Protein expression was analyzed by SDS-PAGE and immunoblot using mouse monoclonal antibodies against TfR1, ORF2 protein or β-actin. (B) Effect of TfR1 knockdown on HEV particle production in PHH. Under the same conditions as in (A), PHH were transfected with nt, Tsg101- or TfR1-targeting siRNAs, followed by full-length HEV p6 RNA transfection and baricitinib treatment. Intra- and extracellular infectivities were measured by focus-forming unit (FFU) assays 3 days post-transfection and are shown relative to the nt control. Data represent the mean of three independent experiments performed in triplicate. Statistical analysis was performed using two-way ANOVA followed by Dunnett’s multiple comparison test. ** P < 0.005; *** P < 0.0001.

To validate these observations in a physiological cell model, PHH were used. Efficient replication of full-length HEV RNA in PHH was achieved by treatment with the Jak inhibitor baricitinib blocking the intrinsic interferon response, as previously described [21] (S7 Fig). Under these conditions, siRNA-mediated TfR1 silencing was combined with transfection of full-length HEV p6 RNA to assess the role of TfR1 in virus production. As shown in Fig 6A, immunoblotting confirmed the efficiency of siRNA-mediated silencing of TfR1 expression in PHH, with a 75% decrease in normalized signal intensity as assessed by densitometry analysis. The level of the intracellular as well as of the extracellular ORF2 protein, mainly representing the glycosylated form, was not altered by TfR1 silencing (Fig 6A), suggesting no impact on HEV RNA replication. However, intra- and extracellular infectious HEV titers were decreased by approximately 30% (Fig 6B). Tsg101 silencing resulted in a stronger decrease in intra- and extracellular virus titers (50% and 70%, respectively; Fig 6B).

**Fig 7:**
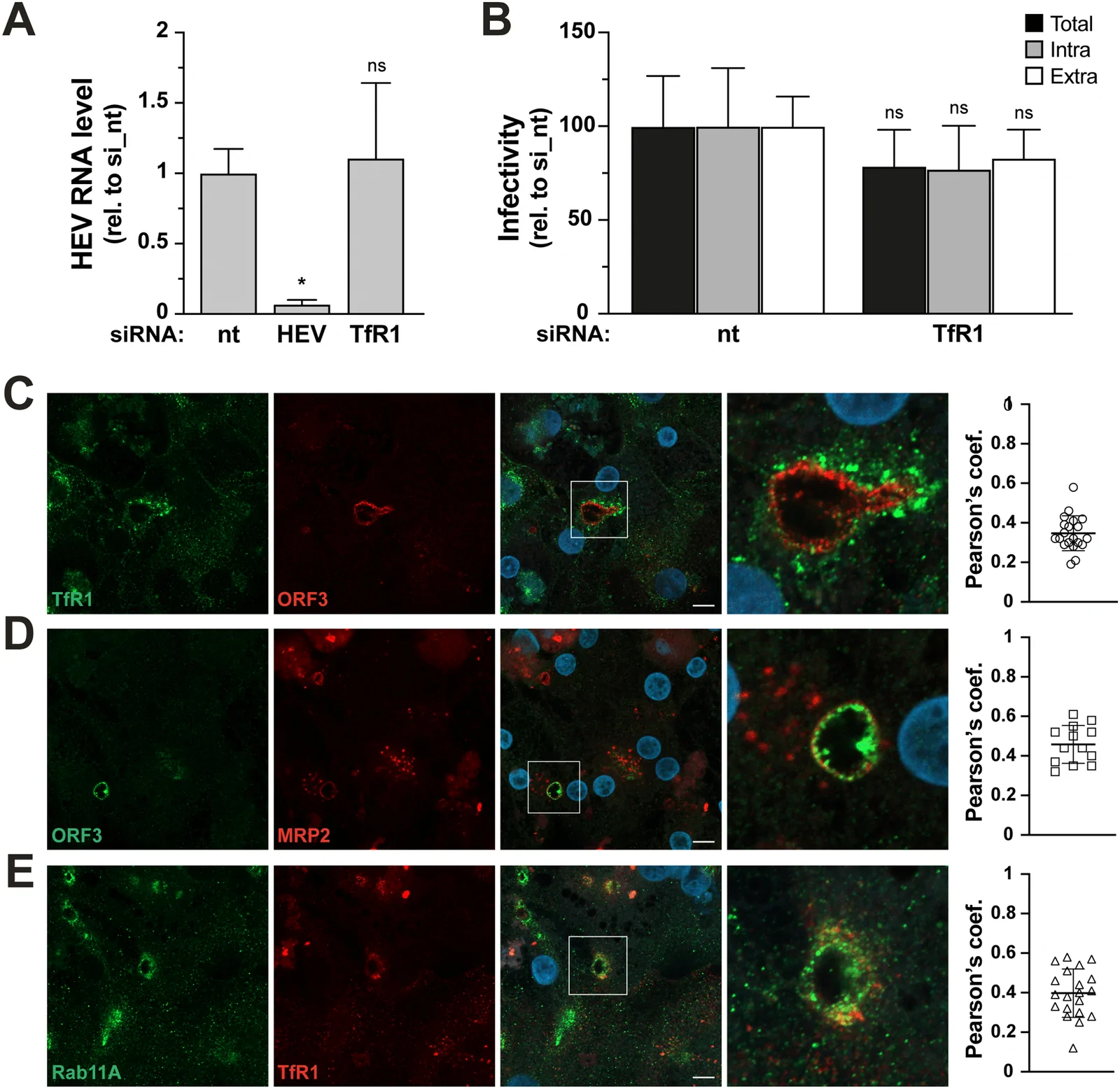
TfR1 knockdown affects HEV production after infection of primary human hepatocytes. (A, B) Effect of siRNA-mediated TfR1 knockdown following HEV infection in primary human hepatocytes (PHH). Cells were transfected with siRNAs targeting TfR1 or the HEV genome (HEV), or with non-targeting (nt) siRNA as control, and infected the following day with HEV p6 particles. (A) HEV RNA levels were quantified by RT-qPCR in samples transfected with nt, HEV or TfR1 siRNAs. Statistical analysis was performed using unpaired nonparametric Mann-Whitney test. ns, not significant; * P < 0.05. (B) Intra- and extracellular infectivities were determined by focus-forming unit (FFU) assays 3 days post-transfection and are shown relative to the nt control. Data represent the mean of two independent experiments performed in triplicate. Statistical analysis was performed using two-way ANOVA followed by Sidak’s multiple comparison test. ns, not significant. (C-E) Immunofluorescence analysis of viral or cellular protein localization in HEV-infected PHH. Three days post-infection, cells grown on collagen-coated coverslips were fixed and stained with (C) mouse monoclonal anti-TfR1 (green) and rabbit polyclonal anti-ORF3 (red), (D) rabbit polyclonal anti-ORF3 (green) and mouse monoclonal anti-MRP2 (red), or (E) rabbit polyclonal anti-Rab11A (green) and mouse monoclonal anti-TfR1 (red). Nuclei were counterstained with DAPI (blue). Representative images, including merged and enlarged views (white squares), are shown. Scale bars, 10 µm. Pearson’s coefficient was determined in at least 13 cells in each condition.

Finally, the role of TfR1 was assessed by gene silencing in PHH after HEV infection. Because modulation of TfR1 expression could affect viral entry, we first quantified HEV RNA in these experimental settings. HEV RNA levels were similar in infected cells transfected with siRNA targeting TfR1 *vs*. a non-targeting control, indicating that virus entry and replication were not detectably affected (Fig 7A). In contrast, intra- and extracellular infectivities were reduced by about 20%. Although this reduction did not reach statistical significance, the trend supports a contribution of TfR1 to efficient virus production in the context of a complete HEV infection cycle (Fig 7B). While this model system is likely to reflect physiological infection, limitations inherent to primary cell culture, including variability in cell viability, infection rates and siRNA transfection efficiency, may contribute to experimental variability and thereby reduce statistical power.

To further corroborate these findings, we investigated the localization of TfR1 and the ORF3 protein in PHH replicating the HEV genome. Interestingly, ORF3 displayed a ring-like structure between adjacent cells that was surrounded by TfR1 staining, although partial colocalization was observed (Fig 7C). To determine the nature of this structure, ORF3 staining was combined with detection of the bile transporter multidrug resistance-associated protein 2 (MRP2), which is expressed at the apical membrane of hepatocytes. Confocal microscopy analysis revealed predominant colocalization of the HEV ORF3 protein with this bile canalicular marker (Fig 7D). We therefore hypothesized that PHH form bile canaliculi-like structures to which HEV ORF3 protein preferentially localizes, consistent with previous reports describing ORF3 at the apical side of polarized cells [15,22]. Similar structures and staining patterns were observed in low-passage Hep293TT cells which can spontaneously organize into rosette-like structures. Upon HEV genome replication, ORF3 protein exhibited a ring-like staining pattern that overlapped with MRP2, suggesting the formation of bile canaliculi-like structures comparable to those observed in PHH (S8 Fig). Moreover, in these cells, TfR1 was also detected in close proximity to ORF3 protein but did not show strong colocalization.

To further investigate TfR1 localization in PHH, double immunostaining for TfR1 and the recycling endosome marker Rab11A was performed, revealing strong colocalization in the area surrounding bile canaliculi-like structures (Fig 7E), consistent with our previous observations in liver-derived cell lines.

Taken together, our proteomics-based approach revealed three novel ORF3-interacting host factors and supports a functional role for TfR1 in HEV production, particularly during the virus assembly step.

## Discussion

Despite its small genome and a simple organization, HEV remains poorly characterized at the molecular level. To advance our understanding of the HEV life cycle, and in particular of the ORF3 protein, we performed a proteomic analysis to identify host interaction partners of this viral protein. Among the candidates tested, four were validated as *bona fide* interactors of ORF3 protein, including the previously described ESCRT-I component Tsg101. Functional analyses using siRNA-mediated silencing indicated that all validated factors may contribute to virus production, with a particularly prominent role for TfR1. Notably, TfR1 colocalized with ORF3 and ORF2 proteins at recycling endosomes, and its functional involvement in infectious HEV production was further confirmed in PHH.

Taking advantage of recombinant monoclonal anti-ORF3 antibodies developed by our group [18], we efficiently immunoprecipitated HEV ORF3 protein together with interacting partners subsequently identified by MS. The use of a ΔORF3 viral construct as a negative control was essential to exclude nonspecific candidates. Interestingly, our screen identified a distinct set of candidate interactors from those reported in previous studies using heterologous two-hybrid systems [23,24]. This limited concordance likely stems from key methodological differences, including the use of hepatic cells, authentic HEV replication, and ORF3 immunoprecipitation in the present study. Importantly, Tsg101 was among the highest-ranking hits in both our dataset and prior reports, providing independent support for the robustness of our approach. Tsg101 interacts with HEV ORF3 protein through a conserved PSAP motif located in the C-terminal region of the viral protein, and this interaction is required for the secretion of infectious HEV particles [11-13]. Consistent with previous studies using ΔORF3 genomes [11,12,25], we observed a marked reduction in virus production upon Tsg101 silencing. Interestingly, this was accompanied by a concomitant increase in HEV RNA replication, reaching about 4-fold for the HEV p6 clone.

As component of the ESCRT-I complex, Tsg101 participates in cargo sorting from early to late endosomes. It has been shown previously that Tsg101 silencing leads to the accumulation of early endosomal membranes as multicisternal structures [26]. In a recent whole genome CRISPR-Cas9 screen, we demonstrated a critical role for early endosomes in HEV RNA replication and showed that perturbation of the endosomal pathway modulates viral genome replication [27]. We therefore hypothesize that Tsg101 silencing may promote HEV RNA replication by altering early endosome homeostasis.

Several candidates identified in the ORF3 pull-down were involved in the regulation of extracellular exosome assembly and, more broadly, the exosomal pathway, as indicated by gene ontology analysis. This finding supports, using an unbiased proteomic approach, that the HEV ORF3 protein acts at the level of exosomes, likely facilitating virion membrane envelopment. Some proteomic hits, such as Alix (PDCD6IP) and Syntenin-1 (SDCB1), are ESCRT-associated proteins acting downstream of Tsg101. Although we did not detect a direct interaction with ORF3 under our experimental conditions, these factors may nonetheless contribute to HEV production as components of the ESCRT machinery, acting in concert with Tsg101.

Among the validated ORF3 protein interaction partners, we identified the poorly characterized proteins CYSTM1 and Rab24, whose silencing reduced HEV production. CYSTM1, also known as C5orf32 or ORF1-FL49, has been described as a marker in several cancers, but its cellular function remains poorly understood [28,29]. Of note, CYSTM1 resembles HEV ORF3 protein in size (97 aa) and the presence of a cysteine-rich region at its C-terminal end. Rab24 is an atypical Rab GTPase with a key role in autophagosome maturation [30]. In mice, Rab24 has also been linked to mitochondrial fission and activity in the liver, correlating with modulation of autophagic flux [31]. Rab24 has additionally been proposed to regulate endosomal degradation through interactions with the late endosome protein Rab7 and its effector RILP (Rab7 interacting lysosomal protein) [32]. The decrease in virus production observed upon Rab24 silencing may therefore be related to its roles in autophagy and late endosome regulation, although further studies will be required to clarify the underlying mechanisms.

The third validated ORF3 protein interaction partner, TfR1, has been extensively studied, especially for its role in cellular iron uptake. Beyond its metabolic function, TfR1 has been described as entry factor for several viruses, including arenaviruses and influenza A virus (reviewed in [33]). The highly pathogenic New World arenaviruses use TfR1 as an entry receptor [34], a feature that constitutes an important determinant of their zoonotic transmission [35]. Influenza A virus can also exploit the TfR1 recycling pathway during cell entry [36,37]. However, to our knowledge, TfR1 has not been implicated in post-entry steps of viral life cycles, making our findings original in demonstrating a role for TfR1 in virion production.

TfR1, which is present at the basolateral membrane of polarized cells, is internalized by clathrin-mediated endocytosis upon binding to transferrin-bound iron. Iron release occurs in endosomes, after which the transferrin-TfR1 complex is transported back to the plasma membrane *via* recycling endosomes [33]. TfR1 trafficking is further regulated by kinase-dependent signaling pathways that control recycling to the plasma membrane *vs*. targeting to late endosomes and lysosomes for degradation [38]. In our study, TfR1 colocalized with ORF3 and ORF2 proteins at recycling endosomes. These observations are consistent with findings by Bentaleb *et al*. [39], who reported the presence of HEV particles on recycling endosomal membranes and proposed a role for the endocytic recycling compartment (ERC) in virion assembly. These authors further showed that the AP-1 complex is involved in directing the infectious form of the ORF2 protein to the ERC [40]. Together with our results, these findings suggest that TfR1 may contribute to virus assembly at the ERC.

We also observed ORF3 localization in PHH at structures resembling bile canaliculi, as indicated by the apical marker MRP2. Similar observations were made in low-passage Hep293TT cells [41], which divide slowly and spontaneously organize into rosette-like structures, rendering them closer to PHH than other liver cancer cell lines such as Hep293TT_AD or S10-3. Under these conditions, ORF3 protein no longer predominantly colocalized with TfR1, which remained associated with recycling endosomes as shown by Rab11A staining. Nevertheless, TfR1 was detected at MRP2-positive bile canaliculi-like structures corresponding to the apical membrane. Similar apical localization of ORF3 has been described in polarized HepG2/C3A F2 cells [15,22], as well as in human liver chimeric mice infected with HEV [42] and in liver biopsies from patients with hepatitis E [43], where ORF3 protein was detected at bile canaliculi. Importantly, the absence of virus shedding into bile upon infection of chimeric mice with ΔORF3 HEV further supports a critical role for ORF3 protein at the apical membrane [15].

Together with our functional data, our findings support a model in which TfR1 participates in HEV assembly at recycling endosomes prior to virus secretion. In this model, the ORF3 protein physically interacts with TfR1 at the site of virus assembly before contributing to the wrapping of HEV particles into exosomal membranes, ultimately allowing their release at the apical membrane.

Altogether, our study advances understanding of the HEV ORF3 protein by identifying three novel host interaction partners. Among these, TfR1 emerges as an important factor for virus production at recycling endosomes, a cellular compartment previously implicated in HEV assembly. These findings contribute to fill an important gap in our knowledge of host factors required for efficient completion of the HEV life cycle.

## Materials and methods

### Cell culture

Hep293TT human hepatoblastoma cells [41] were kindly provided by Gail E. Tomlinson (University of Texas Health Science Center at San Antonio, TX). The cell-culture adapted population Hep293TT_AD was obtained spontaneously after > 30 passages of the parental Hep293TT line. Both cell lines were cultured in RPMI 1640 medium containing 25 mM HEPES buffer, 2 mM L-glutamine supplemented with 10% inactivated fetal bovine serum (FBS), and 0.25 mg/mL gentamicin (all from Gibco, Thermo Fischer Scientific). S10-3 [44] and Huh-7.5 [45] human hepatocellular carcinoma cells were kindly provided by Suzanne U. Emerson (National Institutes of Health, Bethesda, MD) and Charles M. Rice (The Rockefeller University, New York, NY), respectively. Cells were maintained at 37°C in a humidified incubator with 5% CO_2_.

### Reagents

Mouse monoclonal antibodies (mAbs) against β-actin and the FLAG tag, rabbit mAb against FLAG and rabbit polyclonal antibody (pAb) against TfR1 were from Sigma-Aldrich. Rabbit anti-Rab11A pAb and mouse anti-TfR1 mAb were from Cell Signaling Technology and BD Biosciences, respectively. Mouse mAbs against CYSTM1, Rab24 and MRP2 were from Santa Cruz Biotechnology. Recombinant mouse mAbs against the ORF3 protein were described previously [18]. Rabbit anti-ORF3 pAb was from Bioss Antibodies. Mouse mAb 1E6 against HEV ORF2 protein was from Millipore and rabbit anti-ORF2 pAb was kindly provided by Rainer G. Ulrich (Friedrich Loeffler Institute, Riems, Germany). Alexa Fluor-conjugated secondary antibodies were from Thermo Fisher Scientific. Horseradish peroxidase-conjugated secondary antibodies were from Thermo Fisher Scientific. The Jak inhibitor baricitinib was from LubioScience (Zurich, Switzerland).

### Plasmids

Primers used in this study are listed in S1 Table. All constructs were verified by sequencing (Microsynth AG, Balgach, Switzerland). The HEV gt 3 infectious clones HEV83-2-27 (referred to as HEV83–2) [46] and Kernow_C1 p6 (referred to as p6) [47] were kindly provided by Koji Ishii and Takaji Wakita (National Institute of Infectious Diseases, Tokyo, Japan) and by Suzanne U. Emerson (NIH, Bethesda, MD), respectively. Plasmids pUCHEV83–2_ΔORF3 and pUCHEV83–2_Gluc were described in [25]. p6_ΔORF3 was kindly provided by Viet Loan Dao Thi (University of Heidelberg, Germany).

The lentiviral vector pWPI-X-FLAG was described previously [46]. pWPI-FLAG-X was prepared after *Pme*I-*Mlu*I digestion of the pWPI-T7-BLR plasmid (kind gift of Volker Lohmann, University of Heidelberg, Germany) and homologous recombination by Gibson assembly (New England Biolabs) of a DNA fragment generated by touchdown annealing and elongation of primers FLAG-X-fd and FLAG-X-rv.

Lentiviral vectors pWPI-TfR1-FLAG, pWPI-FLAG-CYSTM1 and pWPI-FLAG-Rab24, allowing expression of FLAG-tagged proteins, were prepared by PCR amplification using primer couples TFRC-fd/TFRC-rv, CYSTM1-fd/CYSTM1-rv and Rab24-fd/Rab24-rv with cDNAs MHS6278–202827888, MHS6278–202839762 and MHS6278–202831560 (Horizon Discovery), respectively, as templates, followed by Gibson assembly using the PCR products and *Sma*I-digested pWPI-X-FLAG and pWPI-FLAG-X.

pWPI-mCherry was described previously [46]. Lentiviral vectors pWPI-TfR1-mCherry, pWPI-mCherry-CYSTM1 and pWPI-mCherry-Rab24, allowing expression of mCherry-tagged proteins, were prepared by PCR amplification from pWPI-TfR1-FLAG, pWPI-FLAG-CYSTM1 and pWPI-FLAG-Rab24 using primer couples pWPI-TfR1-cherry-fd/pWPI-TfR1-cherry-rv, pWPICherry-CYSTM1-fd/pWPICherry-CYSTM1-rv and pWPICherry-Rab24-fd/pWPICherry-Rab24-rv, respectively, followed by Gibson assembly using the PCR products and *Sna*BI-digested pWPI-mCherry for TfR1 or *Pme*I-digested pWPI-mCherry for the CYSTM1 and Rab24.

The LentiCRISPRv2 vector used to generate KO cell populations by CRISPR-Cas9 genome editing was a gift from Feng Zhang (Addgene plasmid # 52961) [48]. According to the published cloning strategy, gene-specific guide RNA sequences were inserted into LentiCRISPRv2 by ligation of annealed oligonucleotides (S1 Table) into *Bsm*BI-digested vector backbone, yielding LentiCRISPR_CYSTM1, LentiCRISPR_Rab24 and LentiCRISPR_TfR1.

### Mass spectrometry analysis

Washed magnetic beads were resuspended in 40 μl SDS-PAGE denaturing buffer (2% SDS [w/v], 5% [v/v] beta-mercaptoethanol, 0.1% [w/v] bromophenol blue, 25% Glycerol [v/v], 50 mM Tris-HCl pH 6.8) and heated at 95°C for 5 min. Supernatants were collected and loaded on a 12% mini polyacrylamide gel, run for 3 cm, and stained with Coomassie. Gel lanes between 10–300 kDa were excised into five pieces and digested with sequencing-grade trypsin as described [49]. Extracted tryptic peptides were dried and resuspended in 0.05% trifluoroacetic acid and 2% (v/v) acetonitrile.

Tryptic peptide mixtures were injected into an Ultimate RSLC 3000 nanoHPLC system interfaced *via* a nanospray Flex source to a high resolution QExactive Plus mass spectrometer (Thermo Fisher Scientific). Peptides were first loaded onto an Acclaim PepMap100 C18 trapping microcolumn (20 mm x 100 μm ID, 5 μm, Thermo Fisher Scientific) and then separated on a C18 custom packed column (75 μm ID × 45 cm, 1.8 μm particles; ReproSil-Pur, Dr. Maisch) on a gradient from 4 to 90% acetonitrile in 0.1% formic acid for peptide separation at a flow rate of 250 nl/min (total time 65 min). Full MS survey scans were performed at 70,000 resolution. In data-dependent acquisition controlled by Xcalibur software (Thermo Fisher Scientific), the 10 most intense multiply charged precursor ions detected in the full MS survey scan were selected for higher energy collision-induced dissociation (normalized collision energy 27%) and analysis in the orbitrap at 17,500 resolution. The window for precursor isolation was of 1.5 m/z units around the precursor and selected fragments were excluded for 60 s from further analysis.

MS data were analyzed using Mascot 2.6.2 (Matrix Science) set up to search the human subset of the SWISSPROT database (www.uniprot.org, release of January 2019, 20,368 sequences) supplemented with the sequence of the protein of interest, and a custom contaminant database containing the most usual environmental contaminants and enzymes used for digestion (keratins, trypsin, etc). Trypsin was specified as the proteolytic enzyme (cleavage at K and R), allowing up to two missed cleavages. Mascot was searched with a parent ion tolerance of 10 ppm and a fragment ion mass tolerance of 0.02 Da. Carbamidomethylation of cysteine was specified in Mascot as a fixed modification. Protein N-terminal acetylation and methionine oxidation were specified as variable modifications.

Scaffold (version 4.9.0, Proteome Software) was used to validate MS/MS-based peptide and protein identifications. Peptide identifications were accepted if they could be established at > 90.0% probability by the Percolator posterior error probability algorithm [50]. Protein identifications were accepted if they could be established at > 95.0% probability and required at least two identified peptides. Protein probabilities were assigned using the Protein Prophet algorithm [51]. Proteins that sharing peptides that could not be uniquely assigned based on MS/MS evidence alone were grouped according to the principle of parsimony. Proteins with shared significant peptide evidence were clustered.

### Gene silencing

ON-TARGETplus SMARTPool siRNAs against Tsg101 and TFRC and non- targeting control siRNA were purchased from Dharmacon (Horizon Discovery). HEV-specific siRNA, targeting the ORF1 methyltransferase domain of HEV83–2 or p6 [27], were synthesized by Microsynth AG. Hep293TT_AD cells were seeded at 5 × 10^4^ cells per well in 24-well plates (luciferase assay) or at 2.5 × 10^5^ cells per well in 6-well plates (virus production assays) and transfected with 10 or 8 nM siRNA, respectively, using Lipofectamine RNAiMAX (Qiagen) according to the manufacturer’s instructions.

### In vitro transcription

HEV RNAs were prepared by *in vitro* transcription using the mMESSAGE mMACHINE kit (Ambion, Thermo Fisher Scientific) as described previously [52]. Plasmids were linearized using *Hind*III (HEV 83–2-derived plasmids) or *Mlu*I (HEV p6-derived plasmids).

### Cell transfection and cell electroporation

Five µg of purified *in vitro*-transcribed RNA were electroporated into 6 × 10^6^ Hep293TT or Hep293TT_AD cells using cytomix buffer and a BTX ECM830 electroporator (Harvard Bioscience), as described previously [52]. Electroporated cells were seeded into either 15-cm dishes (co-immunoprecipitation), 6-well plates (virus production) or 24-well plates containing glass coverslips (immunofluorescence).

S10-3 and HepG2/C3A cells were seeded in 24-well plates containing glass coverslips and transfected with 1 µg of purified RNA using the TransIT-mRNA transfection kit (Mirus Bio) according to the manufacturer’s instructions.

### Primary human hepatocytes

PHH (lots CHM2221-HE-C and CHM2225-HE-Z) were purchased from PRIMACYT Cell Culture Technology GmbH as cryopreserved hepatocytes and thawed according to the manufacturer’s instructions. PHH were seeded at 2.2x10^5^ cells per well on collagen-coated 24-well plates (PRIMACYT) or on collagen-coated coverslip (Neuvitro) and maintained in Human Hepatocyte Maintenance Medium (PRIMACYT) at 37°C and 5% CO_2_. At 1 day post-plating, PHH were transfected with siRNA (25 nM) using Lipofectamine RNAiMAX (Qiagen). The next day, PHH were either transfected with HEV RNA using TransIT-mRNA transfection kit (Mirus Bio) or infected with cell culture-derived HEV. PHH were pre-treated with baricitinib 2 µM for 1h at 37°C prior transfection with 500 ng of purified RNA. Medium was changed at 3 days post-plating. Samples for infectivity, immunofluorescence, immunoblot and RT-qPCR were collected at 5 days post-plating.

### Virus production

To prepare inoculum for PHH infection, 6 × 10^6^ S10-3 cells were electroporated with 5 µg *in vitro*-transcribed full-length HEV p6 wt RNA and harvested 7 days post-transfection. Intracellular virus was prepared by pelleting cells, performing three freeze-thaw cycles, centrifugation at 10,000 x g for 30 min, and supernatant collection.

### Focus-forming assay

Focus-forming assays were performed to determine intra- and extracellular viral titers. Intracellular virus was prepared by three freze-thaw cycles followed by centrifugation at 10,000 x g for 30 min; extracellular virus was collected from supernatants. Huh-7.5 cells (4 × 10^4^) were seeded onto coverslips in 24-well plates and infected 24 h later in DMEM without FBS. At 24 h post-infection, medium was replaced with complete DMEM. Cells were fixed at day 5 post-infection with 4% paraformaldehyde for 10 min at room temperature and stained using rabbit anti-ORF2 pAb and Alexa Fluor 488-conjugated secondary antibody. Focus-forming units (FFU) were quantified by fluorescence microscopy (Leica).

### Luciferase assay

Gaussia luciferase activity was measured in supernatant from cells transfected with Gluc replicons derived from the HEV83–2 or p6 clone. Supernatants were collected daily and stored at 4°C until measurement. Luciferase activity was measured by adding 60 µL coelenterazine substrate (0.8 µM) to 10 µL sample and integrating luminescence for 1 s using a GloMax 20/20 luminometer (Promega).

### Indirect immunofluorescence

Hep293TT_AD or Hep293TT cells were fixed at 5 or 6 days post-electroporation, respectively, with 4% PFA for 10 min at room temperature, washed three times with PBS, permeabilized with PBS-0.1% Triton X or PBS-0.5% saponin for 10 min, and blocked with PBS-3% BSA 3% (± 0.05% saponin) for 30 min. Primary antibodies diluted in blocking buffer were incubated for 1 h at room temperature, followed by three PBS washes and incubation with secondary antibodies for 45 min in the dark. Nuclei were counterstained with DAPI, and coverslips were mounted using ProLong Antifade Mountant (Thermo Fisher Scientific). Imaging was performed on a Zeiss LSM 900 Airyscan 2 confocal microscope.

For PHH, fixation was 30 min with 4% PFA, permeabilization was with PBS-0.2% Triton X-100 for 4 min, and blocking was with PBS-5% goat serum for 1 h. Primary antibodies were incubated overnight at 4°C.

The degree of colocalization was quantified by calculating Pearson’s coefficient using ImageJ software.

### Co-immunoprecipitation and immunoblotting

Cells were lysed 5 days after electroporation in immunoprecipitation lysis buffer (50 mM Tris pH7.4, 1 mM EDTA, 150 mM NaCl, 0.2% dodecyl-β-D-maltoside, 1x Complete protease inhibitor). Lysates were cleared by centrifugation at 14,000 x g for 10 min at 4˚C, incubated for 1 h at 4°C with rotation, and centrifuged again. Fifty μL were reserved as input. Remaining supernantants were incubated overnight at 4°C with Dynabeads Protein G (Life Technologies) bound to anti-ORF3 mAb, adjusted to 6 mL with lysis buffer. Beads were washed three times with PBS-0.02% Tween and eluted in 40 µL Laemmli buffer (60 mM Tris·HCl pH 6.8, 25% glycerol, 2% SDS, 0.1% bromophenol blue, 14.4 mM β-mercaptoethanol), heated at 95°C for 10 min, and analyzed by immunoblotting as described previously [53].

### Lentivirus production and cell transduction

Lentiviruses were produced by polyethylenimine (Polysciences) cotransfection of HEK293T cells with pMD2G VSV-G and psPAX2, both kindly provided by Didier Trono (Ecole Polytechnique Fédérale de Lausanne, Switzerland) as well as with pWPI or LentiCRISPR vectors. Medium was replaced by 293 SFM II 16 h post-transfection (Thermo Fisher Scientific). Supernatants were collected at 48 h, filtered (0.45 µm), and used to transduce Hep293TT or Hep293TT_AD cells seeded at 2 × 10^5^ cells per well in 6-well plates. Three days later, cells were transferred to 10-cm dishes and selected for 7 days with either blasticidin (1.5 or 8 µg/mL, respectively) for pWPI-transduced cells or puromycin (5 µg/mL) for LentiCRISPR-transduced cells.

### Quantitative RT-PCR

Total RNA was extracted from HEV-infected PHH using the Quick-RNA kit (Zymo Research). cDNA was synthesized using the High Capacity cDNA Reverse Transcription kit (Applied Biosystems). Quantitative PCR was performed with HEV-specific primers and probes [54] using TaqMan Universal Master Mix II (Applied Biosystems) and a QuantStudio 3 cycler (Thermo Fisher Scientific). Results were normalized to *PGK1* expression, determined using the primers and probe *Hs00943178g1* (Applied Biosystems).

### Statistical analyses

Significance values were calculated by applying unpaired non parametric Mann-Whitney test or two-way ANOVA followed by either Dunnett’s or Sidak’s multiple comparison test with the GraphPad Prism 9 software package (GraphPad Software).

### Use of artificial intelligence

The authors used ChatGPT 5.2 (OpenAI) to assist with proofreading and minor language editing. No content generation, data analysis, or scientific interpretation was performed by the artificial intelligence tool. The authors take full responsibility for the content and final wording of the manuscript.

## Supporting information

S1 Table Primers used in the study. (DOCX)

S2 Table Candidate host proteins identified by mass spectrometry following HEV ORF3 protein pull-down. For each candidate, the protein name, accession number, short identifier (ID), molecular weight (MW) and peptide counts in the analyzed samples (wild-type [wt] or ΔORF3) are indicated. Candidates were selected based on the following criteria: (i) identification by at least three peptides in the wild-type condition and (ii) absence of peptide detection in the ΔORF3 control. All candidates but APOB and IPO9 (*) were further investigated by co-immunoprecipitation.
(DOCX)

S1 Fig HEV ORF3 protein co-immunoprecipitates with Tsg101. Hep293TT_AD cells stably overexpressing FLAG-tagged Tsg101 were electroporated with wt or ΔORF3 full-length HEV83–2 RNA and protein lysates were subjected to ORF3 immunoprecipitation as performed in Fig 1. Protein lysates (Input) as well as immunoprecipitates (IP) were separated onto SDS-PAGE followed by Western-blot analysis using mouse monoclonal anti-FLAG M2 or rabbit polyclonal anti-ORF3 antibodies.
(TIF)

S2 Fig Replication of HEV p6-Gluc replicon in Hep293TT_AD cells. Effect of siRNA-mediated knockdown of the newly identified ORF3-partners on HEV replication assessed by Gluc replicon. Hep293TT_AD cells were electroporated with *in vitro* transcribed HEVp6 Gluc replicon RNA and transfected with siRNA targeting either the HEV RNA, Tsg101, CYSTM1, Rab24 or TfR1 as well as with non-targeting siRNA (nt) as control. Supernatant was collected over 5 days and medium was changed every day. Relative light units (RLU) in culture supernatants were measured at day 1–5 (d1-5) post-electroporation. Results represent the mean of 3 independent experiments.
(TIF)

S3 Fig Co-immunoprecipitation and immunofluorescence detection of endogenous TfR1 in Hep293TT_AD cells replicating HEV 83–2 clone. (A) Hep293TT_AD cells were electroporated with wt or ΔORF3 full-length HEV83–2 RNA and protein lysates were subjected to ORF3 immunoprecipitation as performed in Fig 1B. Protein lysates (Input) as well as immunoprecipitates (IP) were separated onto SDS-PAGE followed by Western-blot analysis using mouse monoclonal anti-TfR1 or rabbit polyclonal anti-ORF3 antibodies. (B) Subcellular localization of TfR1 and of HEV viral proteins. Hep293TT_AD cells were electroporated with *in vitro* transcribed full-length HEV83–2 wild-type (wt) and ΔORF3 RNAs. Five days post-electroporation, cells grown on glass coverslips were fixed and subjected to immunofluorescence with mouse monoclonal antibody against TfR1 [red] and rabbit polyclonal anti-ORF3 [green] (B) or anti-ORF2 antibodies [green] (C). Nuclei were counterstained with DAPI (blue). Representative images including with merge are shown. Scale bars, 10 µm.
(TIF)

S4 Fig Immunofluorescence detection of endogenous TfR1 in Hep293TT_AD cells replicating HEV p6 clone. (A, B) Hep293TT_AD cells were electroporated with *in vitro* transcribed full-length HEV p6 wt or ΔORF3 RNAs. Five days post-electroporation, cells grown on glass coverslips were fixed and subjected to immunofluorescence with mouse monoclonal antibody against TfR1 [red] and rabbit polyclonal anti-ORF3 [green] (A) or anti-ORF2 antibodies [green] (B). Nuclei were counterstained with DAPI [blue]. Representative images including with merge are shown. Scale bars, 10 µm.
(TIF)

S5 Fig Immunofluorescence detection of endogenous TfR1 in S10-3 cells replicating HEV 83–2 clone. (A, B) S10-3 cells, derived from Huh-7, were electroporated with *in vitro* transcribed full-length HEV83–2 wt or ΔORF3 RNAs. Five days post-electroporation, cells grown on glass coverslips were fixed and subjected to immunofluorescence with mouse monoclonal antibody against TfR1 [red] and rabbit polyclonal anti-ORF3 [green] (A) or anti-ORF2 antibodies [green] (B). Nuclei were counterstained with DAPI [blue]. Representative images including with merge are shown. Scale bars, 10 µm.
(TIF)

S6 Fig Virus production of HEV p6 clone following TfR1 expression silencing in S10-3 cells. Effect of the siRNA-mediated TfR1 knockdown on HEV p6 production. S10-3 cells were electroporated with *in vitro* transcribed full-length HEV p6 RNA (n = 6) and transfected with siRNA targeting HEV or TfR1 as well as with non-targeting siRNA (nt) as control. Intra- and extracellular infectivities were measured by focus-forming unit (FFU) determination 5 days post-electroporation. Total, as well as intra- and extracellular infectivities are shown relative to nt control. Statistical analysis was performed by two-way ANOVA followed by Dunnett’s multiple comparison test. * P < 0.05; *** P < 0.0005.
(TIF)

S7 Fig Effect of baricitinib in primary human hepatocytes (PHH) transfected with HEV RNA. PHH were plated in collagen-coated wells and transfected with *in vitro*-transcribed full-length HEV p6 RNA 24 h later. PHH were treated or not with the JAK inhibitor baricitinib (2 µM) one hour prior to transfection and the following days. Three days post-transfection, cells were fixed and stained with anti-ORF2 rabbit pAb (green) and nuclei were counterstained with DAPI (blue). Representative merge images are shown. Scale bars, 100 µm.
(TIF)

S8 Fig HEV ORF3 protein is present at bile canaliculi-like structure in low-passage Hep293TT cells. Hep293TT cells stably overexpressing mCherry-tagged TfR1 were electroporated with wt or ΔORF3 full-length HEV83–2 RNA and grown for 5 days on glass coverslips. Following fixation, cells were subjected to immunofluorescence with anti-ORF3 rabbit polyclonal antibodies (green) and anti-MRP2 mouse monoclonal antibody (grey). TfR1 is visualized by intrinsic mCherry fluorescence (red). Nuclei were counterstained with DAPI (blue). Representative images including with merge and zoom are shown. Scale bars, 10 µm.
(TIF)

## References

[1] Kamar N, Izopet J, Pavio N, Aggarwal R, Labrique A, Wedemeyer H, et al. Hepatitis E virus infection. Nat Rev Dis Primers. 2017;3:17086. doi: 10.1038/nrdp.2017.86 29154369

[2] European Association for the Study of the Liver. EASL clinical practice guidelines on hepatitis E virus infection. J Hepatol. 2018;68(6):1256–71. doi: 10.1016/j.jhep.2018.03.005 29609832

[3] Rein DB, Stevens GA, Theaker J, Wittenborn JS, Wiersma ST. The global burden of hepatitis E virus genotypes 1 and 2 in 2005. Hepatology. 2012;55(4):988–97. doi: 10.1002/hep.25505 22121109

[4] Purdy MA, Drexler JF, Meng XJ, Norder H, Okamoto H, Van der Poel WHM. ICTV virus taxonomy profile: hepeviridae 2022. J Gen Virol. 2022;103(9). 10.1099/jgv.0.001778 PMC12642825 36170152

[5] Debing Y, Moradpour D, Neyts J, Gouttenoire J. Update on hepatitis E virology: implications for clinical practice. J Hepatol. 2016;65(1):200–12. doi: 10.1016/j.jhep.2016.02.045 26966047

[6] Nimgaonkar I, Ding Q, Schwartz RE, Ploss A. Hepatitis E virus: advances and challenges. Nat Rev Gastroenterol Hepatol. 2018;15(2):96–110. doi: 10.1038/nrgastro.2017.150 29162935 PMC11329273

[7] Feng Z, Hensley L, McKnight KL, Hu F, Madden V, Ping L, et al. A pathogenic picornavirus acquires an envelope by hijacking cellular membranes. Nature. 2013;496(7445):367–71. doi: 10.1038/nature12029 23542590 PMC3631468

[8] Feng Z, Hirai-Yuki A, McKnight KL, Lemon SM. Naked viruses that aren’t always naked: quasi-enveloped agents of acute hepatitis. Annual Review of Virol. 2014;1(1):539–60. 10.1146/annurev-virology-031413-085359 PMC12175490 26958733

[9] Nagashima S, Jirintai S, Takahashi M, Kobayashi T, Tanggis, Nishizawa T, et al. Hepatitis E virus egress depends on the exosomal pathway, with secretory exosomes derived from multivesicular bodies. J Gen Virol. 2014;95(Pt 10):2166–75. doi: 10.1099/vir.0.066910-0 24970738

[10] Glitscher M, Hildt E. Hepatitis E virus egress and beyond - the manifold roles of the viral ORF3 protein. Cell Microbiol. 2021;23(12):e13379. doi: 10.1111/cmi.13379 34272798

[11] Emerson SU, Nguyen HT, Torian U, Burke D, Engle R, Purcell RH. Release of genotype 1 hepatitis E virus from cultured hepatoma and polarized intestinal cells depends on open reading frame 3 protein and requires an intact PXXP motif. J Virol. 2010;84(18):9059–69. doi: 10.1128/JVI.00593-10 20610720 PMC2937629

[12] Nagashima S, Takahashi M, Jirintai, Tanaka T, Yamada K, Nishizawa T, et al. A PSAP motif in the ORF3 protein of hepatitis E virus is necessary for virion release from infected cells. J Gen Virol. 2011;92(Pt 2):269–78. doi: 10.1099/vir.0.025791-0 21068219

[13] Kenney SP, Pudupakam RS, Huang Y-W, Pierson FW, LeRoith T, Meng X-J. The PSAP motif within the ORF3 protein of an avian strain of the hepatitis E virus is not critical for viral infectivity in vivo but plays a role in virus release. J Virol. 2012;86(10):5637–46. doi: 10.1128/JVI.06711-11 22438540 PMC3347299

[14] Nagashima S, Takahashi M, Jirintai S, Tanaka T, Nishizawa T, Yasuda J, et al. Tumour susceptibility gene 101 and the vacuolar protein sorting pathway are required for the release of hepatitis E virions. J Gen Virol. 2011;92(Pt 12):2838–48. doi: 10.1099/vir.0.035378-0 21880841

[15] Sari G, Zhu J, Ambardekar C, Yin X, Boonstra A, Feng Z, et al. The viral ORF3 protein is required for hepatitis E virus apical release and efficient growth in polarized hepatocytes and humanized mice. J Virol. 2021;95(23):e0058521. doi: 10.1128/JVI.00585-21 34523963 PMC8577391

[16] Tyagi S, Korkaya H, Zafrullah M, Jameel S, Lal SK. The phosphorylated form of the ORF3 protein of hepatitis E virus interacts with its non-glycosylated form of the major capsid protein, ORF2. J Biol Chem. 2002;277(25):22759–67. doi: 10.1074/jbc.M200185200 11934888

[17] Ding Q, Heller B, Capuccino JMV, Song B, Nimgaonkar I, Hrebikova G, et al. Hepatitis E virus ORF3 is a functional ion channel required for release of infectious particles. Proc Natl Acad Sci U S A. 2017;114(5):1147–52. doi: 10.1073/pnas.1614955114 28096411 PMC5293053

[18] Gouttenoire J, Pollán A, Abrami L, Oechslin N, Mauron J, Matter M, et al. Palmitoylation mediates membrane association of hepatitis E virus ORF3 protein and is required for infectious particle secretion. PLoS Pathog. 2018;14(12):e1007471. doi: 10.1371/journal.ppat.1007471 30532200 PMC6307819

[19] Dong C, Zafrullah M, Mixson-Hayden T, Dai X, Liang J, Meng J, et al. Suppression of interferon-α signaling by hepatitis E virus. Hepatology. 2012;55(5):1324–32. doi: 10.1002/hep.25530 22183878

[20] Xu L-D, Zhang F, Miao C, Yu X, Zhu Y, Zhang M-D, et al. pORF3-driven biogenesis of lipid droplets facilitates HEV infectivity. Cell Rep. 2025;44(10):116406. doi: 10.1016/j.celrep.2025.116406 41066231

[21] Kinast V, Andreica I, Ahrenstorf G, Gömer A, Elsner C, Schlienkamp S, et al. Janus kinase-inhibition modulates hepatitis E virus infection. Antiviral Res. 2023;217:105690. doi: 10.1016/j.antiviral.2023.105690 37517633

[22] Capelli N, Marion O, Dubois M, Allart S, Bertrand-Michel J, Lhomme S, et al. Vectorial release of hepatitis E virus in polarized human hepatocytes. J Virol. 2019;93(4):e01207-18. doi: 10.1128/JVI.01207-18 30463960 PMC6364016

[23] Corneillie L, Lemmens I, Weening K, De Meyer A, Van Houtte F, Tavernier J, et al. Virus-host protein interaction network of the hepatitis E virus ORF2-4 by mammalian two-hybrid assays. Viruses. 2023;15(12):2412. doi: 10.3390/v15122412 38140653 PMC10748205

[24] Geng Y, Yang J, Huang W, Harrison TJ, Zhou Y, Wen Z, et al. Virus host protein interaction network analysis reveals that the HEV ORF3 protein may interrupt the blood coagulation process. PLoS One. 2013;8(2):e56320. doi: 10.1371/journal.pone.0056320 23418552 PMC3571956

[25] Oechslin N, Da Silva N, Szkolnicka D, Cantrelle F-X, Hanoulle X, Moradpour D, et al. Hepatitis E virus RNA-dependent RNA polymerase is involved in RNA replication and infectious particle production. Hepatology. 2022;75(1):170–81. doi: 10.1002/hep.32100 34387882 PMC9300124

[26] Doyotte A, Russell MRG, Hopkins CR, Woodman PG. Depletion of TSG101 forms a mammalian “Class E” compartment: a multicisternal early endosome with multiple sorting defects. J Cell Sci. 2005;118(Pt 14):3003–17. doi: 10.1242/jcs.02421 16014378

[27] Oechslin N, Da Silva N, Ankavay M, Moradpour D, Gouttenoire J. A genome-wide CRISPR/Cas9 screen identifies a role for Rab5A and early endosomes in hepatitis E virus replication. Proc Natl Acad Sci U S A. 2023;120(52):e2307423120. doi: 10.1073/pnas.2307423120 38109552 PMC10756275

[28] Zhang N, Zhen J, Kong X. CYSTM1 is a potential diagnostic and prognostic biomarker and correlated with immune infiltrates in hepatocellular carcinoma. J Gastrointest Oncol. 2025;16(5):2245–61. doi: 10.21037/jgo-2025-353 41220728 PMC12598356

[29] Peng Y, Yang J, Ao J, Li Y, Shen J, He X, et al. Single-cell profiling reveals the intratumor heterogeneity and immunosuppressive microenvironment in cervical adenocarcinoma. Elife. 2025;13:RP97335. doi: 10.7554/eLife.97335 40066698 PMC11896611

[30] Ylä-Anttila P, Eskelinen E-L. Roles for RAB24 in autophagy and disease. Small GTPases. 2018;9(1–2):57–65. doi: 10.1080/21541248.2017.1317699 28463543 PMC5902223

[31] Seitz S, Kwon Y, Hartleben G, Jülg J, Sekar R, Krahmer N, et al. Hepatic Rab24 controls blood glucose homeostasis via improving mitochondrial plasticity. Nat Metab. 2019;1(10):1009–26. doi: 10.1038/s42255-019-0124-x 32694843

[32] Amaya C, Militello RD, Calligaris SD, Colombo MI. Rab24 interacts with the Rab7/Rab interacting lysosomal protein complex to regulate endosomal degradation. Traffic. 2016;17(11):1181–96. doi: 10.1111/tra.12431 27550070

[33] Kawabata H. Transferrin and transferrin receptors update. Free Radic Biol Med. 2019;133:46–54. 29969719 10.1016/j.freeradbiomed.2018.06.037

[34] Radoshitzky SR, Abraham J, Spiropoulou CF, Kuhn JH, Nguyen D, Li W, et al. Transferrin receptor 1 is a cellular receptor for New World haemorrhagic fever arenaviruses. Nature. 2007;446(7131):92–6. doi: 10.1038/nature05539 17287727 PMC3197705

[35] Choe H, Jemielity S, Abraham J, Radoshitzky SR, Farzan M. Transferrin receptor 1 in the zoonosis and pathogenesis of New World hemorrhagic fever arenaviruses. Curr Opin Microbiol. 2011;14(4):476–82. doi: 10.1016/j.mib.2011.07.014 21807555 PMC3159852

[36] Mazel-Sanchez B, Niu C, Williams N, Bachmann M, Choltus H, Silva F, et al. Influenza A virus exploits transferrin receptor recycling to enter host cells. Proc Natl Acad Sci U S A. 2023;120(21):e2214936120. doi: 10.1073/pnas.2214936120 37192162 PMC10214170

[37] Wang X, Li Y, Ji D, Wang Y, Wang X, Guo K, et al. TfR1 facilitates influenza virus endocytosis and uncoating by interacting with NA and M1 via extracellular and intracellular domains. PLoS Pathog. 2025;21(10):e1013511. doi: 10.1371/journal.ppat.1013511 41071818 PMC12533913

[38] Cao H, Schroeder B, Chen J, Schott MB, McNiven MA. The endocytic fate of the transferrin receptor is regulated by c-Abl kinase. J Biol Chem. 2016;291(32):16424–37. doi: 10.1074/jbc.M116.724997 27226592 PMC4974358

[39] Bentaleb C, Hervouet K, Montpellier C, Camuzet C, Ferrié M, Burlaud-Gaillard J, et al. The endocytic recycling compartment serves as a viral factory for hepatitis E virus. Cell Mol Life Sci. 2022;79(12):615. doi: 10.1007/s00018-022-04646-y 36460928 PMC9718719

[40] Ferrié M, Alexandre V, Montpellier C, Bouquet P, Tubiana T, Mézière L, et al. The AP-1 adaptor complex is essential for intracellular trafficking of the ORF2 capsid protein and assembly of Hepatitis E virus. Cell Mol Life Sci. 2024;81(1):335. doi: 10.1007/s00018-024-05367-0 39117755 PMC11335258

[41] Chen TT-L, Rakheja D, Hung JY, Hornsby PJ, Tabaczewski P, Malogolowkin M, et al. Establishment and characterization of a cancer cell line derived from an aggressive childhood liver tumor. Pediatr Blood Cancer. 2009;53(6):1040–7. doi: 10.1002/pbc.22187 19637320

[42] Sayed IM, Verhoye L, Cocquerel L, Abravanel F, Foquet L, Montpellier C, et al. Study of hepatitis E virus infection of genotype 1 and 3 in mice with humanised liver. Gut. 2017;66(5):920–9. doi: 10.1136/gutjnl-2015-311109 27006186

[43] Lenggenhager D, Gouttenoire J, Malehmir M, Bawohl M, Honcharova-Biletska H, Kreutzer S, et al. Visualization of hepatitis E virus RNA and proteins in the human liver. J Hepatol. 2017;67(3):471–9. doi: 10.1016/j.jhep.2017.04.002 28412294

[44] Emerson SU, Nguyen H, Torian U, Purcell RH. ORF3 protein of hepatitis E virus is not required for replication, virion assembly, or infection of hepatoma cells in vitro. J Virol. 2006;80(21):10457–64. doi: 10.1128/JVI.00892-06 16928762 PMC1641774

[45] Blight KJ, McKeating JA, Rice CM. Highly permissive cell lines for subgenomic and genomic hepatitis C virus RNA replication. J Virol. 2002;76(24):13001–14. doi: 10.1128/jvi.76.24.13001-13014.2002 12438626 PMC136668

[46] Shiota T, Li T-C, Yoshizaki S, Kato T, Wakita T, Ishii K. The hepatitis E virus capsid C-terminal region is essential for the viral life cycle: implication for viral genome encapsidation and particle stabilization. J Virol. 2013;87(10):6031–6. doi: 10.1128/JVI.00444-13 23468481 PMC3648136

[47] Shukla P, Nguyen HT, Faulk K, Mather K, Torian U, Engle RE, et al. Adaptation of a genotype 3 hepatitis E virus to efficient growth in cell culture depends on an inserted human gene segment acquired by recombination. J Virol. 2012;86(10):5697–707. doi: 10.1128/JVI.00146-12 22398290 PMC3347312

[48] Sanjana NE, Shalem O, Zhang F. Improved vectors and genome-wide libraries for CRISPR screening. Nat Methods. 2014;11(8):783–4. doi: 10.1038/nmeth.3047 25075903 PMC4486245

[49] Shevchenko A, Tomas H, Havlis J, Olsen JV, Mann M. In-gel digestion for mass spectrometric characterization of proteins and proteomes. Nat Protoc. 2006;1(6):2856–60. doi: 10.1038/nprot.2006.468 17406544

[50] Käll L, Storey JD, Noble WS. Non-parametric estimation of posterior error probabilities associated with peptides identified by tandem mass spectrometry. Bioinformatics. 2008;24(16):i42-8. doi: 10.1093/bioinformatics/btn294 18689838 PMC2732210

[51] Nesvizhskii AI, Keller A, Kolker E, Aebersold R. A statistical model for identifying proteins by tandem mass spectrometry. Anal Chem. 2003;75(17):4646–58. doi: 10.1021/ac0341261 14632076

[52] Szkolnicka D, Pollán A, Da Silva N, Oechslin N, Gouttenoire J, Moradpour D. Recombinant hepatitis E viruses harboring tags in the ORF1 protein. J Virol. 2019;93(19):e00459-19. doi: 10.1128/JVI.00459-19 31315997 PMC6744232

[53] Moradpour D, Englert C, Wakita T, Wands JR. Characterization of cell lines allowing tightly regulated expression of hepatitis C virus core protein. Virology. 1996;222(1):51–63. doi: 10.1006/viro.1996.0397 8806487

[54] Fraga M, Doerig C, Moulin H, Bihl F, Brunner F, Müllhaupt B, et al. Hepatitis E virus as a cause of acute hepatitis acquired in Switzerland. Liver Int. 2018;38(4):619–26. doi: 10.1111/liv.13557 28834649

