# Supplementary material for: Transferrin receptor 1 is required for efficient hepatitis E virus production
Source: PLoS Pathog. 2026 Aug 18;22(8):e1014484. doi: 10.1371/journal.ppat.1014484 (PMC13492984; doi:10.1371/journal.ppat.1014484)
Supplement: Supplementary file 1 [file ppat.1014484.s001.docx]

| **Name** | **Sequence (5'-3')** |
| --- | --- |
| X-FLAG-fd | CACGAGACTAGCCTCGAGGTTTACCATGTCCCGGGCGATTACAAGGATGACGACGATAAATGA |
| X-FLAG-rv | CAGCCCGTAGTTTACTAGTACGCGTCATTTATCGTCGTCATCCTTGTAATCGCCCG |
| TSG101-fd | AGGATGACGACGATAAAGGATCCCAGGCGGTGTCGGAGAGCCAGCT |
| TSG101-rv | TTACTAGTACGCGTGCGTCACCCGTAGAGGTCACTGAGACCGG |
| RAB24-fd | AGGATGACGACGATAAAGGATCCCAGAGCGGGCAGCGCGTGGACGT |
| RAB24-rv | TTACTAGTACGCGTGCGTCACCCGTGATGACAACAGCTGTAGAAG |
| TFRC-fd | GCCTCGAGGTTTACCATGTCCCAGATGGATCAAGCTAGATCAGCAT |
| TFRC-rv | GTCGTCATCCTTGTAATCGCCCGAAAACTCATTGTCAATGTCCCAA |
| CYSTM1-fd | AGGATGACGACGATAAAGGATCCCAGAACCAAGAGAACCCTCCACCA |
| CYSTM1-rv | TTACTAGTACGCGTGCGTCACCCGGTGAGCATGTCCCAGAGAC |
| pWPICherry-Tsg101-fd | TGGACGAGCTGTACAAGGGTTTGGGATCCGCGGTGTCGGAGAGCCAGCT |
| pWPICherry-Tsg101-rv | CTAGTACGCGTGCGATCGCCCCTAGTTTAGTAGAGGTCACTGAGACCGG |
| pWPI-TFR1-Cherry-fd | CGAGACTAGCCTCGAGGTTTACCATGATGGATCAAGCTAGATCAG |
| pWPI-TFR1-Cherry-rv | CGCCCTTGCTCACCATGGTACTACCAAACTCATTGTCAATGTCCCA |
| pWPICherry-CYSTM1-fd | GGACGAGCTGTACAAGGGTTTGGGATCCAACCAAGAGAACCCTCCACCA |
| pWPICherry-CYSTM1-rv | CTAGTACGCGTGCGATCGCCCCTAGTTTAGGTGAGCATGTCCCAGAGACA |
| pWPICherry-Rab24-fd | TGGACGAGCTGTACAAGGGTTTGGGATCCAGCGGGCAGCGCGTGGACGTC |
| pWPICherry-Rab24-rv | CTAGTACGCGTGCGATCGCCCCTAGTTTAGTGATGACAACAGCTGTAGAAG |
| gRNA1 CYSTM1-fd | CACCGCAGGAGCCTCCTAAAACCAC |
| gRNA1 CYSTM1-rv | AAACGTGGTTTTAGGAGGCTCCTGC |
| gRNA1 Rab24-fd | CACCGCCGATAGTAGATTCTACTCA |
| gRNA1 Rab24-rv | AAACTGAGTAGAATCTACTATCGGC |
| gRNA3 TFRC-fd | CACCGATCACTATAGATCCATTCAC |
| gRNA3 TFRC-rv | AAACGTGAATGGATCTATAGTGATC |
