# Supplementary material for: Transferrin receptor 1 is required for efficient hepatitis E virus production
Source: PLoS Pathog. 2026 Aug 18;22(8):e1014484. doi: 10.1371/journal.ppat.1014484 (PMC13492984; doi:10.1371/journal.ppat.1014484)
Supplement: Supplementary file 2 [file ppat.1014484.s002.docx]

| **Candidate characteristics** | | | | **Peptide counts** | |
| --- | --- | --- | --- | --- | --- |
| **Protein name** | **Accession number** | **Short ID** | **MW** (kDa) | **wt** | **ΔORF3** |
| **Hepatitis E virus ORF3 protein** | BAM78545.1_ORF3 |  | 11 | **13** | 0 |
| **Apolipoprotein B-100*** | APOB_HUMAN | APOB | 516 | **8** | 0 |
| **Tumor susceptibility gene 101 protein** | TSG101_HUMAN | TSG101 | 44 | **8** | 0 |
| **Protein ABHD14B** | ABHEB_HUMAN | ABHD14B | 22 | **7** | 0 |
| **14-3-3 protein beta/alpha** | 1433B_HUMAN | YWHAB | 28 | **5** | 0 |
| **Ras-related protein Rap-2b** | RAP2B_HUMAN | RAP2B | 21 | **5** | 0 |
| **Importin-9*** | IPO9_HUMAN | IPO9 | 116 | **5** | 0 |
| **Annexin A1** | ANXA1_HUMAN | ANXA1 | 39 | **5** | 0 |
| **Syntenin-1** | SDCB1_HUMAN | SDCBP | 32 | **4** | 0 |
| **Metalloproteinase inhibitor 3** | TIMP3_HUMAN | TIMP3 | 24 | **4** | 0 |
| **NEDD4-like E3 ubiquitin-protein ligase WWP2** | WWP2_HUMAN | WWP2 | 99 | **4** | 0 |
| **Peptidyl-prolyl cis-trans isomerase-like 1** | PPIL1_HUMAN | PPIL1 | 18 | **4** | 0 |
| **Cold shock domain-containing protein E1** | CSDE1_HUMAN | CSDE1 | 89 | **3** | 0 |
| **Programmed cell death 6-interacting protein** | PDC6I_HUMAN | PDCD6IP | 96 | **3** | 0 |
| **Ras-related protein Rab-24** | RAB24_HUMAN | RAB24 | 23 | **3** | 0 |
| **Proteasome subunit alpha type-7** | PSA7_HUMAN (+1) | PSMA7 | 28 | **3** | 0 |
| **PAC3** | PAC3_HUMAN (+1) | PSMG3 | 48 | **3** | 0 |
| **Melanoma-associated antigen D1** | MAGD1_HUMAN | MAGED1 | 86 | **3** | 0 |
| **Transferrin receptor 1** | TFR1_HUMAN | TFRC | 85 | **3** | 0 |
| **Cysteine-rich and TM domain-containing protein 1** | CYSTM1_HUMAN | CYSTM1 | 11 | **3** | 0 |
| **Neutrophil defensin 1** | DEF1_HUMAN (+1) | DEFA1 | 10 | **3** | 0 |
| **Signal peptidase complex catalytic subunit SEC11A** | SC11A_HUMAN | SEC11A | 21 | **3** | 0 |
| **E3 ubiquitin-protein ligase RNF5** | RNF5_HUMAN | RNF5 | 20 | **3** | 0 |
