# Supplementary material for: Transferrin receptor 1 is required for efficient hepatitis E virus production
Source: PLoS Pathog. 2026 Aug 18;22(8):e1014484. doi: 10.1371/journal.ppat.1014484 (PMC13492984; doi:10.1371/journal.ppat.1014484)
Supplement: Supplementary file 3 [file ppat.1014484.s012.docx]

PPATHOGENS-D-26-00488

**Point-by-point reply to the editors' and reviewers' comments**

**Editor comments to authors**

Your study has now been evaluated by three experts in the field, who reached overall positive conclusions. However, two reviewers identified several points that should be addressed to strengthen the manuscript.

While one of the reviewers suggested additional experiments to map the precise interactions between ORF3 and TfR1, we believe that such analyses may extend beyond the scope of the current study unless these data are already available. In contrast, the proposed knockout experiments to validate the roles of TfR1, Rab24, and CYSTM1 in HEV production are considered important and would significantly strengthen your conclusions.

We therefore encourage you to revise the manuscript with particular attention to these points.

We thank the Editor and the reviewers for their positive feedback and insightful comments as well as for the opportunity to revise our manuscript. The comments allowed us to significantly improve the manuscript by, among other revisions, (1) including knockout experiments for TfR1, Rab24 and CYSTM1 (new Figure 4), (2) quantifying colocalization in the immunofluorescence analyses (revised Figures 2, 5 and 7), and (3) complementing the Introduction and Discussion sections.

**Reviewer #1**

The lab group of Gouttenoire and Moradpour performed an elegant set of biochemical studies to characterize host proteins interacting with the HEV ORF3 protein and further delineate the assembly and egress pathway for HEV particles. They confirm and characterize several of their proteomic hits utilizing primary hepatocyte cultures with genomic knockdowns to validate their studies and show additional interesting characteristics such as the possible involvement of bile-canaliculi like structures in the recycling endosome trafficking pathway. I think the article is well written and the studies appear mostly rigorous. Some experimental aberrations could be better accounted for in the discussion as discussed below. I also felt a few articles from the primary literature could improve the overall introduction and be integrated into the discussion.

I don't feel that new experiments are necessary per se but some key items should be addressed prior to acceptance as follows:

1. I think several references to primary literature regarding HEV assembly and egress could be added to the publication either in introduction or discussion sections.

For instance:

https://doi.org/10.1128/jvi.06711-11 which shows involvement of PSAP motif in efficient particle egress and interaction with TSG101.

DOI: 10.1099/vir.0.066910-0 PMID: 24970738 additional work by Nagashima beyond the 2011 citation included by the authors

DOI:10.1038/s41467-025-61071-y an interesting new take on Rab11 vesicles in entry of non enveloped HEV particles

https://doi.org/10.1111/cmi.13379 A solid mini review focused on the role of ORF3 in egress.

doi: 10.1073/pnas.2418751122 Role of annexin 2 in orf3 vesicle sorting

https://doi.org/10.3389/fcimb.2025.1661270 alternative Golgi-associated egress in the absence of pORF3

We thank the reviewer for his/her valuable comments and suggested amendments. We included several of the proposed references in the revised manuscript to better support the statements made in the Introduction and Discussion sections.

2. I’m not sure references 5 and 6 support the magnitude of the sentence statements on lines 89/90 “…the small ORF3 protein is required for viral particle secretion”

Reference 6 states “Thus, the ESCRT pathway would explain how eHEV particles are formed; however, this aspect suggests that non-enveloped HEV uses a different mechanism for release that is thus far unknown,”

Reference 5 is similarly vague on the totality of the MVB and necessity of ORF3 for release.

Some of the references I have suggested for addition would indicate that while ORF3 plays a significant role in particle release that ORF2 release can be detected in the absence of ORF3 function.

As such, I also feel that statements in lines 96-97 “Viral egress involves the multivesicular body pathway and is strictly dependent on the ORF3 protein” could be softened to account for the potential non-totality of ORF3’s involvement in particle release.

Perhaps authors could also circumvent this by using Emerson’s J Virol 2010 paper (Current citation 8) or Sari et al (citation 10) as the reference and conclude that in primary hepatocytes or polarized cells, ORF3 is essential for particle release? Or reference Z Feng’s literature regarding the multitude of forms of ORF2 (DOI: 10.1097/HEP.0000000000000421) that could explain previous studies in which researchers detected ORF2 release in the absence of functional ORF3?

We thank the reviewer for his/her insightful comments. In the revised version of the manuscript, we softened our statement concerning the strict dependence of viral egress on the ORF3 protein. Along with the complements above (cf. point 1 above), we believe that the statements are now better supported by the references.

3. Have the authors used a Pearson’s Correlation Coefficient analysis to confirm colocalization (or FRET) to confirm the imaging results?

We thank the reviewer for this valuable comment. To address this point, we evaluated the colocalization by determining Pearson's coefficients for all immunofluorescence analyses (revised Figures 2, 5 and 7).

4. Figure 5B: Can the authors postulate as to why they do not see intracellular accumulation of infectious particles in the intracellular domain with TfR1 as they do with Tsg101? One would anticipate that simply blocking egress would accumulate infectious particles to the cellular compartments if they were not released.

We agree with the reviewer that intracellular accumulation should theoretically be observed if HEV egress is impaired. However, it is difficult to observe such variations in primary human hepatocytes, as viral particle secretion is inefficient in this system. Indeed, secreted infectious particles represent < 1% of the total infectivity, as reported by Todt D *et al*. (PNAS 2020;117:1731-1741). We therefore believe that any intracellular accumulation of infectious HEV is likely to be difficult to detect reliably when secretion is impaired in this experimental system.

5. Can the authors mention (discussion) how their proteomics screen compares to some of the other reports of ORF3 interacting host proteins? For instance https://doi.org/10.1128/jvi.02571-08 suggests microtubules interact with ORF3, doi: 10.5812/hepatmon.13902 saw an interaction with hepsin, https://doi.org/10.1371/journal.pone.0056320 reported numerous interacting ORF3 partners but many of these do not appear in this screen. Can the authors address these differences in the discussion?

We thank the reviewer for this valuable comment. Indeed, studies performed in heterologous expression systems have suggested several ORF3 protein interaction partners which we did not identify in our screen. These differences likely reflect the distinct experimental systems employed across studies. Of note, however, the different systems consistently identify Tsg101 as interaction partner of the ORF3 protein. We discussed this point and included the corresponding references in the revised Discussion section (L396-400).

**Reviewer #2**

The study advances understanding of the HEV ORF3 protein by identifying three novel host interaction partners. Among these, TfR1 emerges as an important factor for virus production at recycling endosomes, a cellular compartment previously implicated in HEV assembly. These findings contribute to fill a gap in our knowledge of host factors required for efficient completion of the HEV life cycle.

1. Line 95ff: To improve clarity, the authors could specify that virions shed in feces are initially enveloped and that this envelope is likely degraded during passage through the bile duct (e.g., by bile acids). This clarification would help avoid potential misunderstandings regarding particle forms.

We thank the reviewer for this comment which helped us to improve clarity of our manuscript by specifying this important aspect.

2. Line 129: A more detailed explanation of why the ΔORF3 (dORF3) variant was chosen as a control would strengthen the experimental rationale and help readers better appreciate the study design.

We appreciate the reviewer's comment, which allowed us to better explain the rationale for using this HEV construct as a control.

3. Immunoprecipitation experiments: While 293T cells are technically convenient for interaction studies, a brief justification of their use in the context of a liver-tropic virus would be valuable. If feasible, complementary experiments in liver-derived cell lines - or a discussion of potential similarities and differences - could further enhance the physiological relevance of the findings.

We fully agree with the reviewer. All our immunoprecipitation experiments have been conducted in the human hepatoblastoma-derived cell line Hep293TT and not in kidney-derived HEK293T cells, as the reviewer may have misunderstood. We agree that the designation of this liver-derived cell line is misleading, but this is how it was named initially (Chen TT *et al*. Pediatr Blood Cancer 2009;53:1040-1047). We have clarified the origin of Hep293TT cells in both the Results and Materials and Methods sections.

4. Figure 1B: Including ORF2 staining in the input controls would help demonstrate comparable transfection efficiencies between WT and ΔORF3 genomes, as well as across the different overexpression cell lines.

The faint ORF3 signal in the ΔORF3 condition (CYSTM1 OE) is noticeable. Clarifying whether this reflects minor leakiness of the construct or background signal would be helpful for interpretation.

We attempted to perform ORF2 immunoblotting on the original Western blot membranes; however, ORF2 could not be detected on this archived experimental material. Likewise, we attempted to repeat the detection of CYSTM1 in the ΔORF3 construct to obtain a clearer image, but these efforts were unsuccessful.

As the experiments requested by the reviewer would require reproduction of the entire dataset, we were unable to include these additional controls in the revised manuscript.

5. Figure 2: The microscopy data are suggestive of co-localization; adding quantitative analysis (e.g., Pearson’s or Manders’ coefficients) would further substantiate the proposed interaction.

Figure 4: As for Figure 2, quantitative co-localization analysis would enhance the robustness of the microscopy findings.

Figure 6C, D: Quantification of co-localization would make these findings more convincing and allow for clearer comparison between conditions.

We fully agree with this comment which concurs with point 3 raised by Reviewer #1. To address this point, we evaluated the colocalization by determining Pearson's coefficients for all immunofluorescence analyses (revised Figures 2, 5 and 7).

6. Figure 3: The differential effects of CYSTM1 knockdown on strain 83-2 versus p6 are intriguing. Expanding the discussion on why extracellular infectivity is more prominently affected in p6 (similar to the Rab24 phenotype) could provide additional biological insight and highlight potential strain-specific mechanisms.

We acknowledge that the differential effects observed between the two HEV clones are interesting. In our siRNA-mediated knockdown experiments, extracellular infectivity was significantly reduced for the p6 clone but not for the 83-2 clone. However, during the revision process, we obtained additional data from knockout cell populations that did not reproduce these differential effects. Hence, we believe it is more appropriate to avoid overinterpreting clone-specific differences and instead focus our conclusions on the phenotypes that were consistently observed across experimental settings.

7. Line 180–186: If available, demonstrating co-immunoprecipitation of endogenous TfR1 in liver-derived cell lines would significantly strengthen the physiological relevance of the interaction data.

We agree with the reviewer that it is an important information. Indeed, we performed immunoprecipitation of ORF3 followed by detection of endogenous TfR1 in Hep293TT cells electroporated with wt and ΔORF3 HEV RNA. The data are presented in Figure S3A.

8. Figure 5: The observation that extracellular ORF2 protein levels remain similar despite reduced infectivity is interesting. It would be helpful to discuss whether extracellular ORF2 mainly represents glycosylated, non-infectious forms. Indicating the molecular weight differences or using an antibody specific for non-glycosylated ORF2 (as described in recent studies) could clarify the relationship between ORF2 levels and infectivity.

We thank the reviewer for this insightful comment. As previously demonstrated in studies from the groups of L. Cocquerel and Z. Feng (Montpellier C *et al*. Gastroenterology 2018;154:211–223; Yin X *et al*. PNAS 2018;115:4773–4778), the majority of ORF2 protein detected in cell culture supernatants corresponds to glycosylated forms that exhibit a higher apparent molecular weight than the intracellular, non-glycosylated forms of ORF2. Consistent with these reports, the ORF2 species detected in our Western blots predominantly correspond to the glycosylated form, which is not associated with infectious particles. As suggested by the reviewer, we have now included molecular weight markers in the revised Figure 6A and have clarified this point in the manuscript text.

9. Figure 6A: Providing IF staining for ORF2 in PHH would further support the conclusion that viral replication is not substantially affected in this system.

We agree that this would provide useful additional evidence that viral replication is not affected by TfR1 knockdown. We did perform ORF2 staining; however, the images were acquired at high magnification and are therefore not ideally suited to address the reviewer's comment.

**ORF2 protein detection after HEV infection in siRNA-transfected PHH.** PHH transfected with non-targeted (nt) siRNA or siRNA against TfR1 were infected with HEV p6. Three days post-infection, detection of ORF2 protein was carried out using rabbit polyclonal anti-ORF2 antibodies. Nuclei were counterstained by DAPI.

Although these data could provide complementary information, we believe that the RT-qPCR analysis showing no significant change in intracellular HEV RNA levels already provides strong evidence that viral replication is not substantially affected by TfR1 knockdown. For this reason, we did not include the ORF2 staining data in the revised manuscript.

10. Figure 3B, 3C, Fig 6 and Fig. S6:

Please correct the y-axis name “Infectviity” to “Infectivity”.

Thank you for noticing this typographical error. We corrected all figures in the revised manuscript.

11. Line 253: Since the observed effect in PHH appears mild and co-localization was not demonstrated, a short discussion addressing the potential biological relevance and limitations of this model would strengthen the manuscript.

We thank the reviewer for this comment. In the revised manuscript, we have added the following explanation regarding the lack of statistical significance observed in Figure 7: "While this model system is likely to reflect physiological infection, limitations inherent to primary cell culture, including variability in cell viability, infection rates and siRNA transfection efficiency, may contribute to experimental variability and thereby reduce statistical power." (L 331-334).

12. Line 263: The four-fold increase in viral replication upon TSG101 knockdown is a particularly compelling phenotype. Highlighting this finding more prominently in the results section and discussing possible mechanisms would add depth to the study.

We agree with the reviewer that this observation is interesting. We hypothesize that interference with the endosomal pathway is a plausible explanation, as we described earlier that Rab5A and early endosomes play an important role in HEV replication. This hypothesis is discussed in lines 408-414 of the revised manuscript.

13. To further refine the mechanistic understanding, the authors might consider mapping the interaction domains using ORF3 mutants and/or TfR1 mutants, followed by interaction and co-localization assays. This could provide more direct insight into the molecular basis of the observed effects.

We thank the reviewer for this comment. We agree that this question is of interest and may help elucidate the mechanism underlying our findings. However, as also suggested by the Editor, we considered these investigations to be beyond the scope of the present study and therefore did not pursue them in the current revision.

14. To further strengthen the observed effect, the authors should consider performing knockout experiments to validate the roles of TfR1, Rab24, and CYSTM1 in HEV virus production.

We thank the reviewer for this valuable comment. To address this point, we generated Hep293TT_AD cell populations transduced with LentiCRISPR vectors encoding gRNAs targeting TfR1, Rab24, or CYSTM1, thereby establishing knockout cell populations for each gene. Using the same experimental approach as in our study, we electroporated these cells with HEV p6 full-length RNA and quantified viral infectivity in both the intra- and extracellular compartments. These experiments further support the conclusion that loss of TfR1, Rab24, or CYSTM1 impairs virus production. These new data have been incorporated into the revised manuscript as new Figure 4 and are discussed in the Results and Discussion sections.

**Reviewer #3**

This elegant study by Da Silva et al demonstrates that transferrin receptor 1 (TfR1) is an important host factor involved in the production of infectious HEV particles. After MS analysis of ORF3 immunoprecipitated proteins they identified 22 potential ORF3 interactors, of which the interaction with Tsg101, a previously identified ORF3 interactor, and CYSTM1, TfR1 and Rab24 was confirmed by Co-IP using FLAG-tagged proteins; and by IF confocal microscopy.

Using siRNA approaches they showed that both intracellular and extracellular infectivity was reduced, especially after knock-down of TfR1, suggesting their implication in virion assembly. In addition, they showed that TfR1 colocalizes with ORF2 and ORF3 at recycling endosomes. Finally, they confirmed their observation in primary human hepatocyte cultures and showed that ORF3 protein co-localized especially with MRP2 at bile canaliculi-like resembling structures.

Overall, this is a very interesting paper describing the identification of 3 novel host factors that play a role in HEV assembly. The experiments are well designed and the results are convincing. The data presented further increase our understanding of the HEV life cycle and the host factors involved.

We thank the reviewer for his/her very positive assessment and comments.
