# Supplementary material for: Transferrin receptor 1 is required for efficient hepatitis E virus production
Source: PLoS Pathog. 2026 Aug 18;22(8):e1014484. doi: 10.1371/journal.ppat.1014484 (PMC13492984; doi:10.1371/journal.ppat.1014484)
Supplement: Peer review history

# Peer review: Decision Letter 0

**DOI:** 10.1371/journal.ppat.1014484.r001

**Authors:** Alexander Ploss, Ashley St. John

15 Apr 2026

Transferrin Receptor 1 is Required for Efficient Hepatitis E Virus Production

PLOS Pathogens

Dear Dr. Gouttenoire,

Thank you for submitting your manuscript to PLOS Pathogens. After careful consideration, we feel that it has merit but does not fully meet PLOS Pathogens's publication criteria as it currently stands. Therefore, we invite you to submit a revised version of the manuscript that addresses the points raised during the review process.

Please submit your revised manuscript by Jun 14 2026 11:59PM. If you will need more time than this to complete your revisions, please reply to this message or contact the journal office at plospathogens@plos.org. When you're ready to submit your revision, log on to https://www.editorialmanager.com/ppathogens/ and select the 'Submissions Needing Revision' folder to locate your manuscript file.

Please include the following items when submitting your revised manuscript:

* A letter that responds to each point raised by the editor and reviewer(s). You should upload this letter as a separate file labeled 'Response to Reviewers'. This file does not need to include responses to any formatting updates and technical items listed in the 'Journal Requirements' section below.

* A marked-up copy of your manuscript that highlights changes made to the original version. You should upload this as a separate file labeled 'Revised Manuscript with Track Changes'.

* An unmarked version of your revised paper without tracked changes. You should upload this as a separate file labeled 'Manuscript'.

If you would like to make changes to your financial disclosure, competing interests statement, or data availability statement, please make these updates within the submission form at the time of resubmission. Guidelines for resubmitting your figure files are available below the reviewer comments at the end of this letter.

We look forward to receiving your revised manuscript.

Kind regards,

Alexander Ploss, Ph.D.

Academic Editor

PLOS Pathogens

Ashley St. John

Section Editor

PLOS Pathogens

Editor-in-Chief

PLOS Pathogens

orcid.org/0000-0003-2946-9497

Michael Malim

PLOS Pathogens

orcid.org/0000-0002-7699-2064

**Additional Editor Comments:**

Dear Dr. Gouttenoire,

Thank you for submitting your manuscript to PLoS Pathogens. Your study has now been evaluated by three experts in the field, who reached overall positive conclusions. However, two reviewers identified several points that should be addressed to strengthen the manuscript.

While the one of the reviewers suggested additional experiments to map the precise interactions between ORF3 and TfR1, we believe that such analyses may extend beyond the scope of the current study unless these data are already available. In contrast, the proposed knockout experiments to validate the roles of TfR1, Rab24, and CYSTM1 in HEV production are considered important and would significantly strengthen your conclusions.

We therefore encourage you to revise the manuscript with particular attention to these points.

Best wishes,

Alexander Ploss

**Journal Requirements:**

If the reviewer comments include a recommendation to cite specific previously published works, please review and evaluate these publications to determine whether they are relevant and should be cited. There is no requirement to cite these works unless the editor has indicated otherwise.

1) Please ensure that the CRediT author contributions listed for every co-author are completed accurately and in full.

At this stage, the following Authors/Authors require contributions: Nathalie Da Silva, Angela Pollán, Luca Truscello, Manfredo Quadroni, Darius Moradpour, and Jérôme Gouttenoire. Please ensure that the full contributions of each author are acknowledged in the "Add/Edit/Remove Authors" section of our submission form.

The list of CRediT author contributions may be found here: https://journals.plos.org/plospathogens/s/authorship#loc-author-contributions

2) We ask that a manuscript source file is provided at Revision. Please upload your manuscript file as a .doc, .docx, .rtf or .tex. If you are providing a .tex file, please upload it under the item type u2018LaTeX Source Fileu2019 and leave your .pdf version as the item type u2018Manuscriptu2019.

3) Please upload all main figures as separate Figure files in .tif or .eps format. For more information about how to convert and format your figure files please see our guidelines:

https://journals.plos.org/plospathogens/s/figures

4) We notice that your supplementary Figures, and Tables are included in the manuscript file. Please remove them and upload them with the file type 'Supporting Information'. Please ensure that each Supporting Information file has a legend listed in the manuscript after the references list.

5) In the online submission form, you indicated that your data will be submitted to a repository upon acceptance. We strongly recommend all authors deposit their data before acceptance, as the process can be lengthy and hold up publication timelines. Please note that, though access restrictions are acceptable now, your entire minimal dataset will need to be made freely accessible if your manuscript is accepted for publication. This policy applies to all data except where public deposition would breach compliance with the protocol approved by your research ethics board. If you are unable to adhere to our open data policy, please kindly revise your statement to explain your reasoning and we will seek the editor's input on an exemption.

6) Please amend your detailed Financial Disclosure statement. This is published with the article. It must therefore be completed in full sentences and contain the exact wording you wish to be published.

1) State what role the funders took in the study. If the funders had no role in your study, please state: "The funders had no role in study design, data collection and analysis, decision to publish, or preparation of the manuscript."

2) If any authors received a salary from any of your funders, please state which authors and which funders..

If you did not receive any funding for this study, please simply state: u201cThe authors received no specific funding for this work.u201d

7) Some material included in your submission may be copyrighted. According to PLOS's copyright policy, authors who use figures or other material (e.g., graphics, clipart, maps) from another author or copyright holder must demonstrate or obtain permission to publish this material under the Creative Commons Attribution 4.0 International (CC BY 4.0) License used by PLOS journals. Please closely review the details of PLOS's copyright requirements here: PLOS Licenses and Copyright. If you need to request permissions from a copyright holder, you may use the PLOS Content Copyright Permission Form Please respond directly to this email and provide any known details concerning your material's license terms and permissions required for reuse, even if you have not yet obtained copyright permissions or are unsure of your material's copyright compatibility. Once you have responded and addressed all other outstanding technical requirements, you may resubmit your manuscript within Editorial Manager. Potential Copyright Issues: i) Figure 1A. Please confirm whether you drew the images / clip-art within the figure panels by hand. If you did not draw the images, please provide (a) a link to the source of the images or icons and their license / terms of use; or (b) written permission from the copyright holder to publish the images or icons under our CC BY 4.0 license. Alternatively, you may replace the images with open source alternatives. See these open source resources you may use to replace images / clip-art: - https://commons.wikimedia.org - https://openclipart.org/.

8) Please send a completed 'Competing Interests' statement, including any COIs declared by your co-authors. If you have no competing interests to declare, please state "The authors have declared that no competing interests exist". Otherwise please declare all competing interests beginning with the statement "I have read the journal's policy and the authors of this manuscript have the following competing interests"

**Reviewers' Comments:**

Reviewer's Responses to Questions

**Part I - Summary**

Please use this section to discuss strengths/weaknesses of study, novelty/significance, general execution and scholarship.

Reviewer #1: Assessment. The lab group of Gouttenoire and Moradpour performed an elegant set of biochemical studies to characterize host proteins interacting with the HEV ORF3 protein and further delineate the assembly and egress pathway for HEV particles. They confirm and characterize several of their proteomic hits utilizing primary hepatocyte cultures with genomic knockdowns to validate their studies and show additional interesting characteristics such as the possible involvement of bile-canaliculi like structures in the recycling endosome trafficking pathway. I think the article is well written and the studies appear mostly rigorous. Some experimental aberrations could be better accounted for in the discussion as discussed below. I also felt a few articles from the primary literature could improve the overall introduction and be integrated into the discussion.

Reviewer #2: The study advances understanding of the HEV ORF3 protein by identifying three novel host interaction partners. Among these, TfR1 emerges as an important factor for virus production at recycling endosomes, a cellular compartment previously implicated in HEV assembly. These findings contribute to fill a gap in our knowledge of host factors required for efficient completion of the HEV life cycle.

Line 95ff:

To improve clarity, the authors could specify that virions shed in feces are initially enveloped and that this envelope is likely degraded during passage through the bile duct (e.g., by bile acids). This clarification would help avoid potential misunderstandings regarding particle forms.

Line 129:

A more detailed explanation of why the ΔORF3 (dORF3) variant was chosen as a control would strengthen the experimental rationale and help readers better appreciate the study design.

Immunoprecipitation experiments:

While 293T cells are technically convenient for interaction studies, a brief justification of their use in the context of a liver-tropic virus would be valuable. If feasible, complementary experiments in liver-derived cell lines—or a discussion of potential similarities and differences—could further enhance the physiological relevance of the findings.

Figure 1B:

Including ORF2 staining in the input controls would help demonstrate comparable transfection efficiencies between WT and ΔORF3 genomes, as well as across the different overexpression cell lines.

The faint ORF3 signal in the ΔORF3 condition (CYSTM1 OE) is noticeable. Clarifying whether this reflects minor leakiness of the construct or background signal would be helpful for interpretation.

Figure 2:

The microscopy data are suggestive of co-localization; adding quantitative analysis (e.g., Pearson’s or Manders’ coefficients) would further substantiate the proposed interaction.

Figure 3:

The differential effects of CYSTM1 knockdown on strain 83-2 versus p6 are intriguing. Expanding the discussion on why extracellular infectivity is more prominently affected in p6 (similar to the Rab24 phenotype) could provide additional biological insight and highlight potential strain-specific mechanisms.

Line 180–186:

If available, demonstrating co-immunoprecipitation of endogenous TfR1 in liver-derived cell lines would significantly strengthen the physiological relevance of the interaction data.

Figure 4:

As for Figure 2, quantitative co-localization analysis would enhance the robustness of the microscopy findings.

Figure 5:

The observation that extracellular ORF2 protein levels remain similar despite reduced infectivity is interesting. It would be helpful to discuss whether extracellular ORF2 mainly represents glycosylated, non-infectious forms. Indicating the molecular weight differences or using an antibody specific for non-glycosylated ORF2 (as described in recent studies) could clarify the relationship between ORF2 levels and infectivity.

Figure 6A:

Providing IF staining for ORF2 in PHH would further support the conclusion that viral replication is not substantially affected in this system.

Figure 6C, D:

Quantification of co-localization would make these findings more convincing and allow for clearer comparison between conditions.

Figure 3B, 3C, Fig 6 and Fig. S6:

Please correct the y-axis name “Infectviity” to “Infectivity”.

Line 253:

Since the observed effect in PHH appears mild and co-localization was not demonstrated, a short discussion addressing the potential biological relevance and limitations of this model would strengthen the manuscript.

Line 263:

The four-fold increase in viral replication upon TSG101 knockdown is a particularly compelling phenotype. Highlighting this finding more prominently in the results section and discussing possible mechanisms would add depth to the study.

Additional suggestion:

To further refine the mechanistic understanding, the authors might consider mapping the interaction domains using ORF3 mutants and/or TfR1 mutants, followed by interaction and co-localization assays. This could provide more direct insight into the molecular basis of the observed effects.

To further strengthen the observed effect, the authors should consider performing knockout experiments to validate the roles of TfR1, Rab24, and CYSTM1 in HEV virus production.

Reviewer #3: This elegant study by Da Silva et al demonstrates that transferrin receptor 1 (TfR1) is an important host factor involved in the production of infectious HEV particles. After MS analysis of ORF3 immunoprecipitated proteins they identified 22 potential ORF3 interactors, of which the interaction with Tsg101, a previously identified ORF3 interactor, and CYSTM1, TfR1 and Rab24 was confirmed by Co-IP using FLAG-tagged proteins; and by IF confocal microscopy.

Using siRNA approaches they showed that both intracellular and extracellular infectivity was reduced, especially after knock-down of TfR1, suggesting their implication in virion assembly. In addition they showed that TfR1 colocalizes with ORF2 and ORF3 at recycling endosomes. Finally, they confirmed their observation in primary human hepatocyte cultures, and showed that ORF3 protein co-localized especially with MRP2 at bile canaliculi-like resembling structures.

Overall, this is a very interesting paper describing the identification of 3 novel host factors that play a role in HEV assembly. The experiments are well designed and the results are convincing. The data presented further increase our understanding of the HEV life cycle and the host factors involved.

**********

**Part II – Major Issues: Key Experiments Required for Acceptance**

Please use this section to detail the key new experiments or modifications of existing experiments that should be absolutely required to validate study conclusions.

Generally, there should be no more than 3 such required experiments or major modifications for a "Major Revision" recommendation. If more than 3 experiments are necessary to validate the study conclusions, then you are encouraged to recommend "Reject".

Reviewer #1: I don't feel that new experiments are necessary per se but some key items should be addressed prior to acceptance as follows:

1. I think several references to primary literature regarding HEV assembly and egress could be added to the publication either in introduction or discussion sections.

For instance:

https://doi.org/10.1128/jvi.06711-11 Which shows involvement of PSAP motif in efficient particle egress and interaction with TSG101.

DOI: 10.1099/vir.0.066910-0 PMID: 24970738 Additional work by Nagashima beyond the 2011 citation included by the authors

DOI:10.1038/s41467-025-61071-y An interesting new take on Rab11 vesicles in entry of non enveloped HEV particles

https://doi.org/10.1111/cmi.13379 A solid mini review focused on the role of ORF3 in egress.

doi: 10.1073/pnas.2418751122 Role of annexin 2 in orf3 vesicle sorting

https://doi.org/10.3389/fcimb.2025.1661270 alternative Golgi-associated egress in the absence of pORF3

2. I’m not sure references 5 and 6 support the magnitude of the sentence statements on lines 89/90 “…the small ORF3 protein is required for viral particle secretion”

Reference 6 states “Thus, the ESCRT pathway would explain how eHEV particles are formed; however, this aspect suggests that non-enveloped HEV uses a different mechanism for release that is thus far unknown,”

Reference 5 is similarly vague on the totality of the MVB and necessity of ORF3 for release.

Some of the references I have suggested for addition would indicate that while ORF3 plays a significant role in particle release that ORF2 release can be detected in the absence of ORF3 function.

As such, I also feel that statements in lines 96-97 “Viral egress involves the multivesicular body pathway and is strictly dependent on the ORF3 protein” could be softened to account for the potential non-totality of ORF3’s involvement in particle release.

Perhaps authors could also circumvent this by using Emerson’s J Virol 2010 paper (Current citation 8) or Sari et al (citation 10) as the reference and conclude that in primary hepatocytes or polarized cells, ORF3 is essential for particle release? Or reference Z Feng’s literature regarding the multitude of forms of ORF2 (DOI: 10.1097/HEP.0000000000000421) that could explain previous studies in which researchers detected ORF2 release in the absence of functional ORF3?

3.Have the authors used a Pearson’s Correlation Coefficient analysis to confirm colocalization (or FRET) to confirm the imaging results?

4. Figure 5B Can the authors postulate as to why they do not see intracellular accumulation of infectious particles in the intracellular domain with TfR1 as they do with Tsg101? One would anticipate that simply blocking egress would accumulate infectious particles to the cellular compartments if they were not released.

5. Can the authors mention (discussion) how their proteomics screen compares to some of the other reports of ORF3 interacting host proteins? For instance https://doi.org/10.1128/jvi.02571-08 suggests microtubules interact with ORF3, doi: 10.5812/hepatmon.13902 saw an interaction with hepsin, https://doi.org/10.1371/journal.pone.0056320 reported numerous interacting ORF3 partners but many of these do not appear in this screen. Can the authors address these differences in the discussion?

Reviewer #2: (No Response)

Reviewer #3: none

**********

**Part III – Minor Issues: Editorial and Data Presentation Modifications**

Please use this section for editorial suggestions as well as relatively minor modifications of existing data that would enhance clarity.

Reviewer #1: Line 81 “only mildly symptoms” to “only mild symptoms”

Line 98 “recruitement” to “recruitment”

Reviewer #2: (No Response)

Reviewer #3: none

**********

PLOS authors have the option to publish the peer review history of their article (what does this mean?). If published, this will include your full peer review and any attached files.

If you choose “no”, your identity will remain anonymous but your review may still be made public.

**Do you want your identity to be public for this peer review?**  For information about this choice, including consent withdrawal, please see our Privacy Policy.

Reviewer #1: No

Reviewer #2: No

Reviewer #3: No

**Figure resubmission:**

**Reproducibility:**

-->?>

# Author comment: Author response to Decision Letter 1

**DOI:** 10.1371/journal.ppat.1014484.r002

12 Jun 2026

Attachment Submitted filename: PPATHOGENS-D-26-00488_Reply_Letter.docx

# Peer review: Decision Letter 1

**DOI:** 10.1371/journal.ppat.1014484.r003

**Authors:** Alexander Ploss, Ashley St. John

16 Jul 2026

Dear Prof. Gouttenoire,

We are pleased to inform you that your manuscript 'Transferrin Receptor 1 is Required for Efficient Hepatitis E Virus Production' has been provisionally accepted for publication in PLOS Pathogens.

Before your manuscript can be formally accepted you will need to complete some formatting changes, which you will receive in a follow up email. A member of our team will be in touch with a set of requests.

Please note that your manuscript will not be scheduled for publication until you have made the required changes, so a swift response is appreciated.

IMPORTANT: The editorial review process is now complete. PLOS will only permit corrections to spelling, formatting or significant scientific errors from this point onwards. Requests for major changes, or any which affect the scientific understanding of your work, will cause delays to the publication date of your manuscript.

Should you, your institution's press office or the journal office choose to press release your paper, you will automatically be opted out of early publication. We ask that you notify us now if you or your institution is planning to press release the article. All press must be co-ordinated with PLOS.

Thank you again for supporting Open Access publishing; we are looking forward to publishing your work in PLOS Pathogens.

Best regards,

Alexander Ploss, Ph.D.

Academic Editor

PLOS Pathogens

Ashley St. John

Section Editor

PLOS Pathogens

Sumita Bhaduri-McIntosh

Editor-in-Chief

PLOS Pathogens

orcid.org/0000-0003-2946-9497

Michael Malim

Editor-in-Chief

PLOS Pathogens

orcid.org/0000-0002-7699-2064

***********************************************************

Reviewer Comments (if any, and for reference):

Reviewer's Responses to Questions

**Part I - Summary**

Please use this section to discuss strengths/weaknesses of study, novelty/significance, general execution and scholarship.

Reviewer #2: All questions solved.

Reviewer #3: (No Response)

**********

**Part II – Major Issues: Key Experiments Required for Acceptance**

Please use this section to detail the key new experiments or modifications of existing experiments that should be absolutely required to validate study conclusions.

Generally, there should be no more than 3 such required experiments or major modifications for a "Major Revision" recommendation. If more than 3 experiments are necessary to validate the study conclusions, then you are encouraged to recommend "Reject".

Reviewer #2: (No Response)

Reviewer #3: (No Response)

**********

**Part III – Minor Issues: Editorial and Data Presentation Modifications**

Please use this section for editorial suggestions as well as relatively minor modifications of existing data that would enhance clarity.

Reviewer #2: (No Response)

Reviewer #3: (No Response)

**********

PLOS authors have the option to publish the peer review history of their article (what does this mean?). If published, this will include your full peer review and any attached files.

If you choose “no”, your identity will remain anonymous but your review may still be made public.

**Do you want your identity to be public for this peer review?**  For information about this choice, including consent withdrawal, please see our Privacy Policy.

Reviewer #2: No

Reviewer #3: No

# Editor report: Acceptance letter

**DOI:** 10.1371/journal.ppat.1014484.r004

**Authors:** Alexander Ploss, Ashley St. John

Dear Prof. Gouttenoire,

We are delighted to inform you that your manuscript, "Transferrin Receptor 1 is Required for Efficient Hepatitis E Virus Production," has been formally accepted for publication in PLOS Pathogens.

We have now passed your article onto the PLOS Production Department who will complete the rest of the pre-publication process. All authors will receive a confirmation email upon publication.

The corresponding author will soon be receiving a typeset proof for review, to ensure errors have not been introduced during production. Please review the PDF proof of your manuscript carefully, as this is the last chance to correct any scientific or type-setting errors. Please note that major changes, or those which affect the scientific understanding of the work, will likely cause delays to the publication date of your manuscript. Note: Proofs for Front Matter articles (Pearls, Reviews, Opinions, etc...) are generated on a different schedule and may not be made available as quickly.

Soon after your final files are uploaded, the early version of your manuscript, if you opted to have an early version of your article, will be published online. The date of the early version will be your article's publication date. The final article will be published to the same URL, and all versions of the paper will be accessible to readers.

For Research Articles, you will receive an invoice from PLOS for your publication fee after your manuscript has reached the completed accept phase. If you receive an email requesting payment before acceptance or for any other service, this may be a phishing scheme. Learn how to identify phishing emails and protect your accounts at https://explore.plos.org/phishing.

Thank you again for supporting open-access publishing; we are looking forward to publishing your work in PLOS Pathogens.

Best regards,

Sumita Bhaduri-McIntosh

Editor-in-Chief

PLOS Pathogens

orcid.org/0000-0003-2946-9497

Michael Malim

Editor-in-Chief

PLOS Pathogens

orcid.org/0000-0002-7699-2064
